# Association between Food, Beverages and Overweight/Obesity in Children and Adolescents—A Systematic Review and Meta-Analysis of Observational Studies

**DOI:** 10.3390/nu15030764

**Published:** 2023-02-02

**Authors:** Dorthe Dalstrup Jakobsen, Lea Brader, Jens Meldgaard Bruun

**Affiliations:** 1 Steno Diabetes Center Aarhus, Aarhus University Hospital, 8200 Aarhus, Denmark; 2 Department of Clinical Medicine, University of Aarhus, 8200 Aarhus, Denmark; 3 Danish National Center for Obesity, 8200 Aarhus, Denmark; 4 Global Nutrition, Arla Foods amba, 8200 Aarhus, Denmark; 5 Department of Nutrition, Exercise, and Sports, University of Copenhagen, 2200 Copenhagen, Denmark

**Keywords:** nutrition, dietary risk factors, childhood obesity, adiposity, BMI-SDS, weight development

## Abstract

A healthy diet is essential to prevent childhood obesity, however, adherence to a healthy diet is challenging. The aim of this study was to give a comprehensive overview of the literature investigaating associations between food and beverages and overweight/obesity in children and adolescents in order to identify dietary risk factors. A systematic search was performed in four databases and observational studies were included. Meta-analysis was performed using the random effect model. Sixty records met inclusion criteria and 14 different food or beverage categories were identified. A higher intake of sugar-sweetened beverages increased the odds of overweight/obesity by 1.20 (*p* < 0.05) (*n* = 26) and higher intake of fast food increased the odds of overweight/obesity by 1.17 (*p* < 0.05) (*n* = 24). Furthermore, higher intake of meat (OR 1.02, *p* < 0.05 (*n*:7)) and refined grains (OR 1.28, *p* < 0.05 (*n*:3)) was associated with an increased risk of overweight/obesity. In contrast, higher intake of whole grain (OR 0.86, *p* = 0.04 (*n*:5)) and more surprisingly sweet bakery (OR 0.59, *p* < 0.05 (*n*:3)) was associated with a decreased risk of overweight/obesity. In conclusion, a higher intake of sugar-sweetened beverages and a higher intake of fast food was identified as the primary dietary risk factors for overweight/obesity. Future research is needed to strengthen the generalizability of these results.

## 1. Introduction

Childhood obesity is reaching alarming proportions in many countries and due to the complex interplay between genetic, psychosocial, and environmental factors the progress in tackling childhood obesity is challenging [1]. Despite an obvious complexity, a healthy diet is still essential to prevent childhood obesity and the development of obesity-related diseases in adulthood [2]. A healthy diet is characterized as a balanced diet including an adequate intake of whole grains, milk and dairy, fish, fruits, and vegetables whereas a dietary pattern rich in meat, soda, fried food, instant noodles, burgers, and pizza is recognized as being unhealthy and obesogenic [2,3,4]. A recent systematic review also suggested that children and adolescents who adhere to dietary patterns composed of unhealthy foods such as the Western diet are more likely to develop obesity [5]; however, evidence supporting beneficial effects on weight development in children when following a healthy diet is scare [6]. Furthermore, most dietary intervention studies show no consistent effect on changes in body mass index in children [7] and adherence to dietary intervention principles seem to decrease over time [8] which highlights the importance of ensuring that dietary advices are converted into sustained dietary habits.

Therefore, the aim of the present study was to summarize the literature investigating association between food and beverages categories and overweight/obesity in children and adolescents. A comprehensive overview of the current literature within this area will help identify lack of knowledge, provide valuable insight on dietary risk factors, and potentially provide simple strategies to promote healthy weight development in children and adolescents.

## 2. Materials and Methods

### 2.1. Data Sources, Search Strategies, and Search Process

The study was designed according to the Preferred Reporting Items for Systematic Reviews and Meta-analyses guidelines (PRISMA) and registered at PROSPERO (ID number: CRD42020173201) [9].

A systematic search was initiated in PubMed, EMBASE, SCOPUS, and Web of Science in March 2020 and in accordance with the PROSPERO protocol the initial search included both randomized controlled trials and longitudinal and cross-sectional studies. A systematic review and meta-analysis based on eligible randomized controlled trials was finalized in July 2021 and has already been published [10] why this paper focuses on evaluating eligible observational studies (i.e., longitudinal and cross-sectional studies). In August 2022, the systematic search was renewed to identify eligible observational studies published within the last two years.

In all four databases the search block for the population (#1) was combined with each search block for the exposure separately (#2 to #10). The search strategy from PubMed is shown in Table 1.

The study selection process was conducted by the first reviewer (D.J.), and questionable records were sent to the second reviewer (J.B.) to assess eligibility. Any doubts or discrepancies were resolved by discussion with the third reviewer (L.B). All identified records were screened by title and abstract and full-text records were retrieved if the title and abstract met the predefined inclusion criteria. Second all full-texts were read carefully to evaluate the study eligibility.

www.covidence.org was used for review management.

### 2.2. Eligibility Criteria

Records were eligible for inclusion based on the following criteria stratified by participants/population, intervention/exposure and comparison, outcome measures and study design (PICOS) [11].

Participants/population: Records were included if investigating otherwise healthy children or adolescents (mean age between 5 and 18 years of age) with overweight and/or obesity or a mixed population of children or adolescents with normal weight and children or adolescents with overweight/obesity. Records were excluded if examining only non-overweight children or adolescents (ISO-Body Mass Index (BMI) < 25 kg/m^2^), athletes or adolescents who underwent bariatric surgery. Also records investigating children or adolescents with diagnosed non-alcoholic-fatty-liver disease, diabetes, or other comorbidities were excluded.

Intervention/exposure and comparison: Records were included if investigating higher consumption of a certain food or beverage (intervention/exposure) as compared with lower consumption of the same food or beverage (comparator) and if they investigated consumption of single food or beverage categories. Records were excluded if they investigated dietary patterns.

Outcome measures: Records were included if the primary outcome was overweight, overweight/obesity, and obesity categorized by an age-and-sex specific BMI comparable to international standards [12,13,14] and if they reported associations between overweight/obesity and food or beverage consumption as odds ratio (OR) and 95% confidence interval (95% CI). Records were excluded if they were not categorized by an age-and-sex-specific BMI; if they reported associations as correlation coefficients (β), mean (SD), or other; or if they did not adjust for any confounders.

Study design: Cross-sectional studies and longitudinal studies were included if they were written in English and published in peer-reviewed journals from 1 January 1990 until 31 August 2022.

### 2.3. Data Extraction and Coding Decisions

For the meta-analysis ORs and 95% CI for the highest consumption were directly extracted from the included records. Some records provided separate outcome measures for different levels of consumption (e.g., 0–1 cups/day, 1–2 cups/day and ≥2 cups/day). In these records, the lowest consumption was always coded as the reference value (OR = 1) and compared to the highest consumption reported. If necessary, the scale reported by the author was reversed. Some records reported outcome measures separately for overweight and obesity while others reported outcome measures for overweight/obesity combined. Whenever available, the outcome measures for the obesity category were extracted and otherwise the outcome measures for the overweight/obesity category were extracted. Data were extracted by the first reviewer (D.J.) and any doubts or questions concerning data extraction were resolved with the second reviewer (J.B.). Discrepancies were solved in discussion with the third reviewer (L.B.). Data extraction forms were created by the authors in Microsoft Excel and included author (year published), country, age (year), sex, study, dietary instrument, definition of high intake, sample size, adjustments, and study quality assessment.

### 2.4. Risk of Bias Assessment and Study Quality Assessment

The methodical quality of included records was assessed using an adapted version of The Newcastle-Ottawa Scale (NOS) for cross-sectional studies [15]. Representativeness of the sample, sample size, response rate, ascertainment of the exposure, adjustment for confounding factors with total energy intake evaluated as the most important factor, reliability of the outcome determination and appropriateness of statistical analysis was evaluated. A maximum of ten stars could be assigned to each study and more than six stars were considered high quality. Guidelines from The Grading of Recommendation Assessment, Development and Evaluation (GRADE) was used to evaluate study quality of evidence [16].

### 2.5. Statistical Analysis

Meta-analysis was carried out if three or more records investigated consumption of the same food or beverage. Whenever possible, age-divided meta-analysis was carried out investigating children, 5–11 years old and adolescents, 12–18 years old. Due to an assumption of heterogeneity between records e.g., in population, dietary instrument used, and the definition of “high intake”, a random effect-model was used. All meta-analyses were analyzed using a significance level of 0.05. Statistical heterogeneity was evaluated based on the Chi^2^ statistics and reported as a tau^2^ and *I*^2^ value below each forest plot [11]. If the heterogeneity was moderate to high (*I*^2^ > 50%), heterogeneity was investigated using subgroup analysis according to age (5–18 years, 5–11 years, 12–18 years) and funnel plots.

Stata software version 17.0 (StataCorp LLC, College Station, TX, USA) was used to perform meta-analyses.

## 3. Results

### 3.1. Description of Records

**Figure 1:**
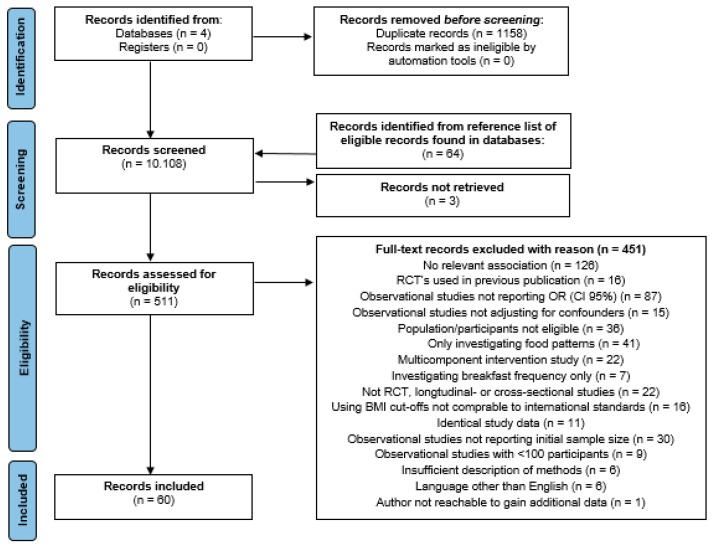
Study selection process.

A total of 10,108 records were identified and 511 full-text records were screened for eligibility. In total, 451 full-text records were excluded due to various reasons of which 87 records were excluded because they did not report OR (95% CI) and 15 records were excluded because reported results were not adjusted for any confounders. Detailed flow-chart is available in Figure 1.

In total, 60 records including 242,061 participants were eligible for data synthesis. Based on the included records 14 different food or beverage categories could be identified. Most records included were cross-sectional studies and few were longitudinal studies. Food and beverage consumption were most often investigated using a self-administrated food-frequency questionnaire, but also one or two-day dietary records, interviews, and other types of questionnaires were used as dietary instrument. Dietary consumption was typically parent-reported for children and self-reported for adolescents (See Supplementary Table S1 for characteristics of included studies).

### 3.2. Synthesis of Results

**Figure 2:**
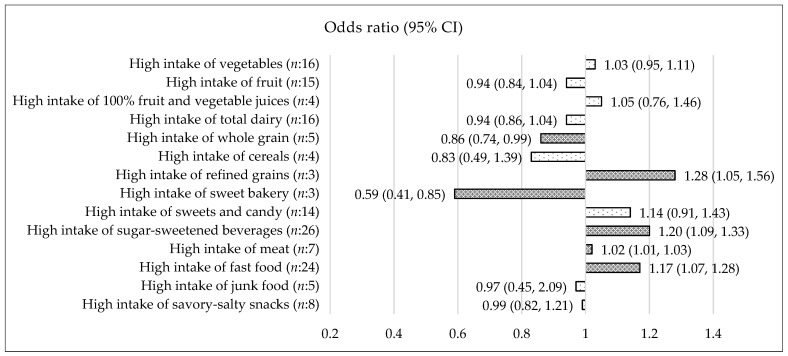
Association between a high intake of 14 different food and beverages categories and risk of overweight/obesity in children and adolescents 5–18 years. OR < 1 = decreased risk of overweight/obesity, OR > 1 = increased risk of overweight/obesity. Dark shades are significant (*p* ≤ 0.05). White shades are not significant. The forest-plots for each food or beverages category is available in Supplementary Figures S1–S15.

**Figure 3:**
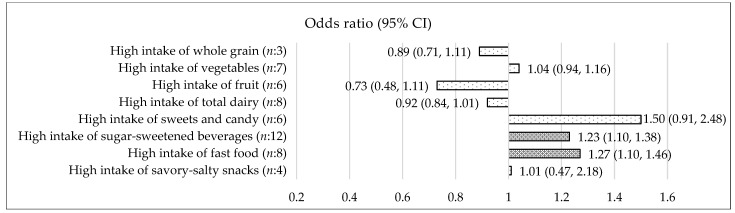
Association between a high intake of eight different food and beverages categories and risk of overweight/obesity in children 5–11 years. OR < 1 = decreased risk of overweight/obesity, OR > 1 = increased risk of overweight/obesity. Dark shades are significant (*p* ≤ 0.05). White shades are not significant. The forest-plots for each food or beverages category is available in Supplementary Figures S16–S24.

**Figure 4:**
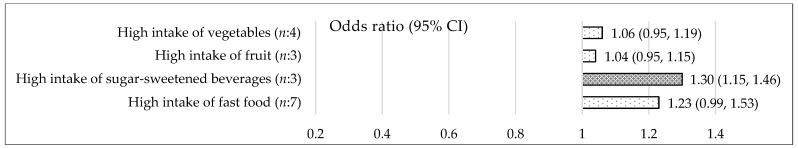
Association between a high intake of four different food and beverages categories and risk of overweight/obesity in adolescents 12–18 years. OR < 1 = decreased risk of overweight/obesity, OR > 1 = increased risk of overweight/obesity. Dark shades are significant (*p* ≤ 0.05). White shades are not significant. The forest-plots for each food or beverages category is available in Supplementary Figures S25–S28.

Vegetables: Based on 16 records (*n*:16) [17,18,19,20,21,22,23,24,25,26,27,28,29,30,31,32], the OR (95% CI) for higher intake of vegetables compared to lower intake of vegetables was 1.03 (0.95, 1.11) (*p* = 0.49) (*I*^2^ = 76.98%) in children and adolescents 5–18 years (Figure 2). In children 5–11 years, the OR (95% CI) was 1.04 (0.94, 1.16) (*p* = 0.43) (*n*:7) (Figure 3) and in adolescents 12–18 years, the OR (95% CI) was 1.06 (0.95, 1.19) (*p* = 0.28) (*n*:4) (Figure 4). The types of vegetables investigated were not specified.

Fruit: Based on 15 records [17,18,19,20,23,25,27,28,29,30,31,33,34,35,36], the OR (95% CI) for higher intake of fruit compared to lower intake of fruit was 0.94 (0.84, 1.04) (*p* = 0.22) (*I*^2^ = 72.44%) in children and adolescents 5–18 years (Figure 2). In children 5–11 years, the OR (95% CI) was 0.73 (0.48, 1.11) (*p* = 0.14) (*n*:6) (Figure 3) and in adolescents 12–18 years, the OR (95% CI) was 1.04 (0.95, 1.15) (*p* = 0.39) (*n*:3) (Figure 4). Types of fruits investigated were not specified.

100% fruit and vegetables juices: Based on four records [29,37,38,39], the OR (95% CI) for higher intake of 100% fruit and vegetable juices versus lower intake of 100% fruit and vegetable juices was 1.05 (0.76, 1.46) (*p* = 0.77) (*I*^2^ = 78.61%) in children and adolescents 5–18 years (Figure 2). 100% fruit and vegetable juices were defined as 100% orange juices, 100% fruit juices/drinks, and fruit/vegetable juice.

Total dairy: Based on 16 records [17,22,25,27,28,39,40,41,42,43,44,45,46,47,48,49], the OR (95% CI) for higher intake of total dairy compared to lower intake of total dairy was 0.94 (0.86, 1.04) (*p* = 0.26) (*I*^2^ = 88.50%) in children and adolescents 5–18 years (Figure 2). Excluding the two outcome measure from the meta-analysis, investigating cheese specifically [22,27] did not change the results (data not shown). In children 5–11 years, the OR (95% CI) for a higher intake of total dairy compared to a lower intake of total dairy was 0.92 (0.84, 1.01) (*p* = 0.08) (*n*:8) (Figure 3). When excluding cheese, the OR (95% CI) for a higher intake of milk and dairy compared to a lower intake of milk and dairy was 0.90 (0.80, 1.00) (*p* = 0.06) (*n*:7) (see Supplementary Figure S20). Total dairy was defined to include white milk, flavored/chocolate milk, dairy and cheese with varying fat content.

Whole grain: Based on five records [17,22,28,50,51], the OR (95% CI) for higher intake of whole grain compared to lower intake of whole grain was 0.86 (0.74, 0.99) (*p* = 0.04) in children and adolescents 5–18 years (Figure 2). In children 5–11 years, the OR (95% CI) was 0.89 (0.71, 1.11) (*p* = 0.30) (*n*:3) (Figure 3). Whole grain was defined as whole grain, grains, bread/grains, and dietary fiber.

Cereals: Based on four records [26,27,40,42], the OR (95% CI) for higher intake of cereals versus lower intake of cereals was 0.83 (0.49, 1.39) (*p* = 0.47) (*I*^2^ = 74.49%) in children and adolescents 5–18 years (Figure 2). Cereals were defined as ready to eat cereal, bread and cereal, porridge, and instant noodles.

Refined grains: Based on three records [24,35,40], the OR (95% CI) for higher intake of refined grains versus lower intake of refined grains was 1.28 (1.05, 1.56) (*p* < 0.05) in children and adolescents 5–18 years (Figure 2). Refined grains were defined as buns, bread foods, or bread and cereals combined.

Sweet bakery: Based on three records [23,26,27], the OR (95% CI) for higher intake of sweet bakery versus lower intake of sweet bakery was 0.59 (0.41, 0.85) (*p* < 0.05) (*I*^2^ = 53.34%) in children and adolescents 5–18 years (Figure 2). Sweet bakery was defined as pastries, cakes, doughnuts, and pies.

Sweets and candy: Based on 14 records [19,22,23,25,26,27,35,38,40,49,52,53,54,55], the OR (95% CI) for higher intake of sweets and candy compared to lower intake of sweets and candy was 1.14 (0.91, 1.43) (*p* = 0.24) (*I*^2^ = 91.95%) in children and adolescents 5–18 years (Figure 2). In children 5–11 years, the OR (95% CI) was 1.50 (0.91, 2.48) (*p* = 0.11) (*n*:6) (Figure 3). Sweets and candy was defined as candy, chocolate, ice cream, sugar, and sweets.

Sugar sweetened beverages: Based on 26 records [17,19,21,22,23,25,29,33,39,40,41,42,43,45,52,53,56,57,58,59,60,61,62,63,64,65], the OR (95% CI) for higher intake of sugar-sweetened beverages versus lower intake of sugar-sweetened beverages was 1.20 (1.09, 1.33) (*p* < 0.05) (*I*^2^ = 79.34%) in children and adolescents 5–18 years (Figure 2). In children 5–11 years, the OR (95% CI) was 1.23 (1.10, 1.38) (*p* < 0.05) (*n*:12) (Figure 3) and in adolescents 12–18 years of age, the OR (95% CI) was 1.30 (1.15, 1.46) (*p* < 0.05) (*n*:3) (Figure 4). Sugar-sweetened beverages were defined as beverages with sugar e.g., soft drinks, sugary beverages, sweetened beverages, and soda. Records investigating 100% fruit/vegetables juices and records including diet drinks were excluded.

Meat: Based on seven records [19,22,27,28,32,40,42], the OR (95% CI) for higher intake of meat compared to lower intake of meat was 1.02 (1.01, 1.03) (*p* < 0.05) in children and adolescents 5–18 years. However, this result was driven primarily by the result from Chen et al. [32] due to a large sample size (Figure 2). When excluding Chen et al. from the meta-analysis, no association between meat intake and overweight/obesity was found (*p* = 0.57) (data not shown). Meat was defined as mixed meats, red meat, meat products, meat and equivalent, meta/fish/eggs, and sausage.

Fast food: Based on 24 records [18,19,20,23,25,27,29,30,31,35,40,42,52,54,55,59,66,67,68,69,70,71,72,73], the OR (95% CI) for higher intake of fast food versus lower intake of fast food was 1.17 (1.07, 1.28) (*p* < 0.05) (*I*^2^ = 56.44) in children and adolescents 5–18 years (Figure 2). In children 5–11 years, the OR (95% CI) was 1.27 (1.10,1.46) (*p* < 0.05) (*n*:8) (Figure 3) and in adolescents 12–18 years, the OR (95% CI) was 1.23 (0.99, 1.53) (*p* = 0.06) (*n*:7) (Figure 4). Fast food was defined as pizza, French fries, burgers, fried potatoes, fast or fatty foods, and fried food.

Junk food: Based on five records [20,24,28,60,74], the OR (95% CI) for higher intake of junk food versus lower intake of junk food was 0.97 (0.45, 2.09) (*p* = 0.94) (*I*^2^ = 86.06%) in children and adolescents 5–18 years (Figure 2). Junk food was defined as high-fat and/or high-sugar foods e.g., sugar-sweetened beverages, pastries, candies, French fries, chips, cakes, and fatty/processed meat.

Savory-salty snacks: Based on eight records [22,23,25,27,29,30,52,53], the OR (95% CI) for higher intake of savory-salty snacks compared to lower intake of savory-salty snacks was 0.99 (0.82, 1.21) (*p* = 0.96) (*I*^2^ = 78.54) in children and adolescents 5–18 years (Figure 2). In children 5–11 years, the OR (95% CI) was 1.01 (0.47, 2.18) (*p* = 0.97) (*n*:4) (Figure 3). Savory-salty snacks were defined as chips/fried potatoes, crisps, popcorn, peanuts, and corn chips.

### 3.3. Risk of Bias Assessment and Study Quality Assessment

Risk of bias assessment using NOS showed that most records were considered high quality (see Supplementary Table S1). However, only few records presented a power calculation and reported a total response rate. Most records did not compare characteristics between respondents and non-respondents, and furthermore, most included records did not adjust for total energy intake, which was evaluated as the most important confounding factor. Due to the study design of included studies, study quality of evidence was evaluated as low to very low for all outcomes [75].

### 3.4. Subgroup Analysis and Publication Bias

Most meta-analysis reported a moderate to high heterogeneity which was investigated using subgroups analysis according to age groups (5–11 years, 12–18 years, 5–18 years) and funnel plots. Only the subgroup analysis investigating sweets and candy showed a significant difference between age groups, which could explain the high heterogeneity reported. Investigating heterogeneity by funnel plots showed that the results concerning fast food consumption, sweets and candy consumption, and fruit consumption could be affected by publication bias. Furthermore, the moderate to high heterogeneity could be due to the different definitions of e.g., cereals, sugar-sweetened beverages and dairy, and the variation in high consumption vs. low consumption between records (see Supplementary Figures S29–S39).

## 4. Discussion

This systematic review and meta-analysis identified higher intake of sugar-sweetened beverages and higher intake of fast food to be the main dietary risk factors for overweight/obesity in children and adolescents 5–18 years of age. In addition, higher intake of meat and a higher intake of refined grains were also associated with an increased risk of overweight/obesity; however, these results were based on only a few records. Only higher intake of whole grain and more surprisingly higher intake of sweet bakery were associated with a decreased risk of overweight/obesity in children and adolescents 5–18 years of age.

Findings from the present study suggest that reducing the intake of sugar-sweetened beverages as well as fast food should be of highest priority to promote healthy weight development among children and adolescents. Based on the present result, a higher intake of sugar-sweetened beverages compared to a lower intake of sugar-sweetened beverages was associated with an increased risk of overweight/obesity in 5–18-year-olds (i.e., 12–18-year-olds and 5–11-year-olds). These results are essentially in line with three previous systematic reviews and meta-analysis [76,77,78]. A way to attenuate the deleterious effects of sugar-sweetened beverages could be to replace these with non-caloric beverages or flavored milk, which seems to have beneficial effects reducing body fat [10]. Currently there is no universal consensus on the definition of sugar-sweetened beverages [79] and in the present meta-analysis, sugar-sweetened beverages include a variety of beverages with sugar. Furthermore, the definition of high intake varies between studies, which altogether might explain the high heterogeneity reported. Nevertheless, these findings and a substantial body of evidence demonstrating a higher intake of sugar-sweetened beverages to increase the risk of overweight/obesity and a higher risk of developing type 2 diabetes, cardiovascular disease, and some cancers [76,77,78,79] show that reducing the intake of sugar-sweetened beverages should be a cornerstone in weight management in children and adolescents.

Based on this systematic review, a higher intake of fast food defined as pizza, French fries, burgers, fried potatoes, fast/fatty foods, and fried food was identified as a dietary risk factor for overweight/obesity in children and adolescents. In children and adolescents, the association between fast food consumption and overweight/obesity is often investigated in relation to fast food access or visits at fast food restaurants [80,81]. In a recent systematic review and meta-analysis, fast food restaurant access was positively associated with fast food consumption. However, only about half of the cohort studies and one-third of the cross-sectional studies reported a positive association between access to fast food restaurants and measures of overweight/obesity and when using BMI-related continuous measures most studies reported no association between access to fast food restaurants and overweight/obesity [81]. Likewise, another systematic review and meta-analysis reported no association between fast food consumption and childhood obesity [76]. The present meta-analysis investigating children and adolescents 5–18 years of age presented with a moderate heterogeneity and investigating the heterogeneity by funnel plots showed that these results could be affected by publication bias. Furthermore, the definition of fast food includes a variety of high-fat foods and several of included studies are not adjusting for total energy intake which altogether could explain the heterogeneity reported. Thus, the evidence supporting a direct association between fast food consumption and overweight/obesity in children and adolescents is scare, and still more high quality evidence is needed.

The results of this systematic review and meta-analysis indicate that a higher intake of meat is associated with an increased risk of overweight/obesity in children and adolescents. However, this result is driven primarily by one study [32] and therefore it must be interpreted with outmost caution. Evidence supporting this association is scare and a recent systematic review and meta-analysis investigating the association between consumption of red meat and overweight/obesity across all ages found no association [82]. In adults, the evidence demonstrating a positive association between meat intake and overweight/obesity is more robust [83,84], but different types of meat (e.g., pork, veal, lamb, red meat) is typically investigated together which could affect the estimate of association.

In the present systematic review and meta-analysis, a higher intake of refined grains was found to be associated with an increased risk of overweight/obesity and in contrast a higher intake of whole grain was associated with a decreased risk of overweight/obesity. These results are essentially in line with the previous studies in adults [84,85] and also in accordance with the current dietary guidelines recommending children, adolescents, and adults to choose whole grain when eating bread foods and cereals [3,4,86]. However, the meta-analysis investigating refined grains was based on only three records and the meta-analysis investigating whole grain was based on only five records why these results must be interpreted with caution. Thus, the evidence supporting these associations in children and adolescents need to be substantiated with more high quality evidence.

To some surprise, a higher intake of sweet bakery defined as pastries, cakes, doughnuts, and pies was found to be associated with a decreased risk of overweight/obesity in children and adolescents. One theory explaining this association could be that sweet bakery is more satiating than sugar from liquids and candy and therefore could be compensating for other calories. However, the most plausible explanation is that the reliability of this result is questionable due to study limitations, e.g., this result is based on only three records, only one of the included studies is adjusting for total energy intake and the meta-analysis presented with a high heterogeneity. Moreover, evidence supporting this association is scare, therefore the result must be interpreted with outmost caution [87]. Hence, added sugar in food should still be minimized and future research investigating children and adolescents needs to investigate the impact of sugar consumption on overweight/obesity and metabolic health in the absence of a positive energy balance.

Based on this systematic review and meta-analysis, higher intake of total dairy was not associated with overweight/obesity in children and adolescents. However, higher intake of milk and dairy (cheese excluded) was non-significantly associated with a decreased risk of overweight/obesity in children 5–11 years (*p* = 0.06). This tendency is in line with results from a recent systematic review and meta-analysis of randomized controlled trials, demonstrating a beneficial effect of higher-dairy diets (600–1000 mL) compared to lower-dairy diets (0–500 mL) by increasing lean body mass and reducing body fat in children and adolescents [10]. Evidence suggests that high intake of dairy is associated with a reduced risk of overweight/obesity in children and adolescents [88,89] and a recent meta-analysis of randomized controlled trials also concluded that children and adolescents consuming milk and milk products were more likely to achieve a lean body phenotype [90]. The present meta-analysis investigating dairy presented with a high heterogeneity possibly due to the different types of dairy investigated. Future research investigating dairy should therefore aim at differentiating between different milk- and cheese-products and also investigate smaller children and older children separately.

Weight management programs do almost always encourage children, adolescents, and their families to eat plenty of fruit and vegetables. However, in the present systematic review and meta-analysis, a higher intake of fruit or vegetables was neither positive nor negatively associated with overweight/obesity in children and adolescents. Fruit and vegetables are often investigated in combination and evidence suggests that diets including fruit and vegetables among other “healthy” foods, are associated with lower likelihood of overweight/obesity in children and adolescents [5,91]. However, for some children and adolescents the consumption of fruit and/or vegetables might be add-on calories which could explain the lack of association. Thus, future research investigating the effect of fruit and vegetables should differentiate between different fruits and vegetables and be conducted in a isocaloric randomized controlled setting.

Results from the present systematic review and meta-analysis must be interpreted in the light of several limitations. First, the study design of the included records limits inference of causality. Second, a small number of records were included in some meta-analysis. Third, only few of the included records adjusted for total energy intake, which is highly relevant and important when investigating associations between diet and weight development. Fourth, different instruments were used to evaluate dietary consumption. Fifth, several of the included records did not provide adequate description of the food or beverages investigated e.g., types of fruit and vegetables and the definition of low versus high consumption varied between records.

## 5. Conclusions

Based on the present systematic review and meta-analysis, a higher intake of sugar-sweetened beverages and a higher intake of fast food was identified as dietary risk factors for overweight/obesity in children and adolescents. Furthermore, a higher intake of meat and a higher intake of refined grains were also associated with an increased risk of overweight/obesity, whereas higher consumption of whole grain and a higher intake of sweet bakery were associated with a decreased risk. Due to a small number of records included in some of the meta-analysis, heterogeneity between records and lack of causality, these results must be interpreted with caution. Nevertheless, this systematic review and meta-analysis underlines that reducing the intake of sugar-sweetened beverages and fast food should be a priority to promote healthy weight development in children and adolescents and furthermore, that future research is needed to investigate associations between consumption of food and beverages and childhood obesity.

## Figures and Tables

**Table 1:** Search strategy used in the systematic literature review.

Population		Exposure
#1 “Body Mass Index”[Mesh] OR “Body Weight Maintenance”[Mesh] OR “Weight Loss”[Mesh] OR “Body Mass Index” OR “Weight Loss” OR “weight maintenance”; AND “child”[Mesh] OR “Adolescent”[Mesh] OR Child* OR adolescen*; AND “Pediatric Obesity”[Mesh] OR “Pediatric Obesity” OR “Childhood Obesity” Sort by: Most Recent	AND	#2 “Carbonated Beverages”[Mesh] OR “Sugar Sweetened Beverages”[Mesh] OR “Beverages”[Mesh] OR “Artificially Sweetened Beverages”[Mesh] OR Beverage* Sort by: Most Recent
		#3 “Vegetables”[Mesh] OR “Fruit”[Mesh] OR “Salads”[Mesh] OR Vegetable* OR Fruit OR Salad* Sort by: Most Recent
		#4 “Dairy Products”[Mesh] OR “Cheese”[Mesh] OR “Yogurt”[Mesh] OR Milk OR Dairy OR Cheese OR Yogurt Sort by: Most Recent
		#5 “Fast Food”[Mesh] OR “Fast Food” Sort by: Most Recent
		#6 “Seafood”[Mesh] OR “Meat”[Mesh] OR “Egg Proteins, Dietary”[Mesh] OR “Poultry”[Mesh] OR Seafood OR Meat OR Egg OR Poultry OR chicken OR fish Sort by: Most Recent
		#7 “Candy”[Mesh] OR “Chocolate”[Mesh] OR “Snacks”[Mesh] OR Candy OR Chocolate OR Snacks OR Cake OR Sweets OR nuts Sort by: Most Recent
		#8 “Whole Grains”[Mesh] OR “Dietary Fiber”[Mesh] OR Grain* OR “Dietary Fiber” Sort by: Most Recent
		#9 potato* OR rice OR pasta Sort by: Most Recent

## Data Availability

Data described in the manuscript, code book and analytic code will be made available upon reasonable request to the corresponding author.

## Supplementary Materials

The following supporting information can be downloaded at: https://www.mdpi.com/article/10.3390/nu15030764/s1. Table S1: Characteristics of included studies and ROB assessment of each study. Figures S1–S15: Individual forest plots (results) for each food and beverage category investigating children and adolescents 5–18 years of age. Figures S16–S24: Individual forest plots (results) for each food and beverage category investigating children and adolescents 5–11 years of age. Figures S25–S28: Individual forest plots (results) for each food and beverage category investigating children and adolescents 12–18 years of age. Figures S29–S39: Subgroup analysis and funnel plots investigating heterogeneity. References [92,93] are cited in the supplementary materials
