# Supplementary material for: Association between Food, Beverages and Overweight/Obesity in Children and Adolescents—A Systematic Review and Meta-Analysis of Observational Studies
Source: Nutrients. 2023 Feb 2;15(3):764. doi: 10.3390/nu15030764 (PMC9920526; doi:10.3390/nu15030764)
Supplement: Supplementary file 1 [file nutrients-15-00764-s001.zip › nutrients-2181573-supplementary.pdf]

# Supplementary Materials: Association Between Food, Beverages and Overweight/Obesity in Children and Adolescents - A Systematic Review and Meta-analysis of Observational Studies

**Table S1:** Characteristics and ROB assessment of included studies

| Author (year published) | Country | Age (yr) | Sex | Study | Dietary instrument | Definition of high intake | Sample size | Adjustments | NOS score |
|---|---|---|---|---|---|---|---|---|---|
| Abreu (2014)[1] | Portugal | 15-18y | Both (separate) | Cross-sectional | FFQ | **Ready to eat cereal:** ≥40 g/d (boys), ≥31 g/d (girls). **Vegetables**: ≥114 g/d (boys). **Sweets/pastries:** ≥57 g/d (girls). | 1209 | Age, maturation, total energy intake (kJ/kcal), low-energy reporters, dietary fiber (g/4184 kJ (1000 kcal)). | 10 |
| Ahmed (2013)[2] | Pakistan | 9-18y | Both | Cross-sectional | FFQ | **Fruit:** ≥4 times per week | 501 | Age, sex and socioeconomic status | 7 |
| Beck (2014)[3] | US (Mexican/American children) | 8-10y | Both | Cross-sectional | YAQ | **Soda, flavored milk, whole milk, 2% milk:** Additional serving of 240 ml | 319 | Age, gender, the retained beverage variables | 7 |
| Bel-Serrat (2019)[4] | Ireland | 6-10y | Both | Longitudinal study (FU) | FFQ | **Fruit:** Every day/most days. **Vegetables**: Every day/most days. **Fast food:** Every day. **Savory snacks:** Every day | 1262 | Measurement round, time follow-up, age, sex, baseline z-BMI, baseline abdominal obesity status, school socio-economic status, school location, and household ownership (rented vs. owned) | 7 |
| Chen (2021)[5] | China | 6-12y | Both | Cross-sectional | FFQ | **Vegetables:** highest %. **Red meat:** highest % | 12813 | Age, gender | 6 |
| Choumenkovitch (2013)[6] | US | 10y (3rd-6th grade) | Both | Cross-sectional | Block food screener (intake and portions size past 24h) | **Whole grain:** >1.5 servings/day | 792 | Age, sex, race/ethnicity, physical activity, state of residence | 7 |
| Colapinto (2014)[7] | Canada | 10-11y (grade 5) | Both | Cross-sectional | The Harward YAQ | **White milk:** ≥ 2 glasses/day. **Chocolate milk:** <1 glass/month | 8958 | Energy intake, sex, region of residence, household income, parental education | 8 |
| Cutler (2012)[8] | US | 12-16y | Both (separate) | Longitudinal study (BA) | YAQ | **Vegetables, fruit:** One quintile increase in dietary pattern factor score. | 3572 | Race/ethnicity, SES, physical activity | 7 |
| Denova-Gutiérrez (2008)[9] | Mexico | 10-19y | Both | Cross-sectional | FFQ | **SSB:** >3 servings/day | 1055 | Age, gender, sexual maturation, place of residence, physical activity, | 9 |

| | | | | | | | | | |
|---|---|---|---|---|---|---|---|---|---|
| | | | | | | | | father's education, total caloric intake, alcohol consumption and energy derived from fat intake | |
| Duan (2020)[10] | China | 11-18y | Both | Cross-sectional | Pediatric Sleep QUA-Sleep-Related Breathing Disorder | **Fruit:** >2 times/week | 1825 | Sex, age, birth history, parental weight, maternal weight, slowness in eating, picky eating | 6 |
| Flores (2013)[11] | US | 5-6y | Both | Longitudinal study (FU) | Interviews (7 day record) | **SSB:** ≥1 times past 7 days. **Fruit:** ≥1 time past 7 days | 6800 | Weighted, forward stepwise procedures used | 7 |
| Gibson (2007)[12] | UK | 7-18y | Both | Cross-sectional | 7-days record (weighed) | **SSB:** >0.55 MJ/day | 1294 | Age, sex, under-reporting, dieting | 8 |
| Govindan (2013)[13] | US | 10-12y | Girls | Cross-sectional | The School Physical Activity and Nutrition QUA | **Milk:** >2 servings in previous 24 hours | 848 | Covariates with p>0.10 in the univariate analysis | 8 |
| Haboush-Deloye (2021)[14] | US | 4-6y | Both | Cross-sectional | 7-day record | **Soda**: Any weekly consumption | 7814 | SES, gender, PA, screen time, feeding practice at 6 months | 6 |
| Hadi (2020)[15] | Indonesia | 7-12y | Both | Cross-sectional | FFQ | **Junk food:** >1050 kcal/d | 488 | Calorie intake, demographic, socio-economic factors | 8 |
| Hanley (2000)[16] | US | 10-19y | Both | Cross-sectional | FFQ | **Vegetables, Junk food, Bread foods:** Fourth quartile ? | 242 | Age, sex | 7 |
| Hatami (2014)[17] | Iran | 10-18y | Both | Cross-sectional | FFQ | **Fruit, vegetables, sweets/candy, soft drinks, SSB, milk, fast food, chips:** 5–7 times/week | 1109 | Age, sex | 7 |
| Heo (2020)[18] | US | Mean age 16y | Both (separate) | Cross-sectional | Youth Risk Behavior Survey | **Soda:** ≥2 times per day | 13.571 | Age, Hispanic ethnicity | 7 |
| Hirschler (2009)[19] | Buenos Aires | 6-12y | Both | Cross-sectional | Interviews (Freq. daily) | **SSB:** >1 glass/day. **Milk:** ≥3 glasses/day | 330 | Fruit and vegetables consumption, milk consumption, maternal educational level, socioeconomic class. | 8 |
| Huus (2009)[20] | Sweden | 5y | Both | Longitudinal study (FU) | FFQ | **Vegetables, fruit, pastries, cereals (porridge), fast food (fried potatoes/French fries), sweets/candy (candy, chocolate, ice-cream), cream/crème fraiche:** Daily. **Chips:** 3-5 times/week. **Cheese:** 3 times/day. **Milk:** 4 times/day or more. **Meat (sausage):** 1-2 times/week. | 5032 | Known risk factors (parental BMI, parental education and heredity for diabetes) | 7 |
| Hwang (2020)[21] | Korea | 10-18y | Both | Cross-sectional | 24h recall | **SSB:** ≥ median consumption (boy:≥280.55 g. girl ≥210 g.) **Fruit/vegetable juices:** ≥ median consump- | 6121 | Age, sex, BMI, household income level, residential area, energy intake | 7 |

| | | | | | | | | | |
|---|---|---|---|---|---|---|---|---|---|
| | | | | | | tion (boy: ≥208 g., girl: ≥187.2 g.) **Milk/milk products:** ≥ median consumption (boy: ≥249.6 g, girl: ≥212 g) | | | |
| Joseph (2015)[22] | India | 12-16y | Only boys | Cross-sectional | FFQ | **Fast food:** Daily or more than daily consumption | 292 | Physical activity | 5 |
| Karki (2019)[23] | Nepal | 6-13y | Both | Cross-sectional | School Physical and Nutrition survey 2010 (past 7 days) | **Soft drinks:** Yes. **Junk food:** ≥2 times/week | 575 | All independent variables | 7 |
| Katzmarzyk (2016)[24] | Australia, Brazil, Canada, China, Columbia, Finland, India, Kenya, Portugal, South Africa, UK, US | 9-11y | Both (separate) | Cross-sectional | FFQ (HBSC) | **Regular soft drinks:** Once a day or more | 6162 | Age, study site, highest parental education, meeting physical activity guidelines | 7 |
| Kollias (2011)[25] | Greece | 6-13y (age-divided) | Both | Cross-sectional | FFQ | **Sweets, fast food:** Yes | 780 | Age | 7 |
| Kostopoulou (2021)[26] | Greece | 10-16y | Both | Cross-sectional | FFQ | **Fast food, sweets:** Frequent consumption | 3504 | Gender, siblings, daily meals, breakfast consumption, consumption of poor-quality food at school | 7 |
| Lee (2018)[27] | South Korea | 9-13y | Both | Longitudinal study (FU) | FFQ | **Fast food:** ≥1 times/week | 833 | Age, sex, BMI | 7 |
| Leon-Guerrero (2020)[28] | Guam (US) | 2-8y | Both | Cross-sectional | 2-day food log | **SSB:** ≥1.09 cups/day | 634 | Community, age, sex, ethnicity | 7 |
| Liu (2012)[29] | US | 2-19y (age-divided) | Both | Cross-sectional | 24 hour dietary interview | **SSB:** ≥24 oz./day. **Whole grain:** ≥1 serving/day. **Vegetables:** ≥1 cup/day. **Fruit:** ≥2 cups/day. **Dairy:** ≥3 cups/day. | 14332 | Age, race/ethnicity, perceived health, household income level, reference person's education, region, survey year, total energy intake. | 10 |
| Maitland (2015)[30] | Turk and Caicos Islands | 10.9 (mean age) | Both | Cross-sectional | FFQ | **Fruit, vegetables, junk food (Miscellaneous):** ≥2 times/day. **Fast food/fried food:** ≥1 times/day. | 297 | Gender, age, nationality, number of years in the Turk and Caicos Islands | 7 |
| Marcos-Pasero (2019)[31] | Spain | 6-9y | Both | Cross-sectional | 48-h food record | **Dairy:** Increase in number of dairy portion/day | 221 | Sex, age | 8 |
| Martinez-Ospina (2019)[32] | Columbia | 7-14y | Both | Cross-sectional | FFQ (HBSC) | **SSB:** >4 days/week. **Fat-free-milk:** Less than once/day or more | 714 | Age, sex, socioeconomic status | 8 |
| Matthews (2011)[33] | US | 6-19y | Both | Cross-sectional | FFQ | **Grains, vegetable, fruit, meat, dairy, junk food:** Highest quartile (Q4) | 1764 | Gender, type of school, soda intake, frequency of consumption of | 8 |

| | | | | | | | | | |
|---|---|---|---|---|---|---|---|---|---|
| | | | | | | | | all of the other six food groups | |
| Mekonnen (2018)[34] | Ethiopia | 6-12y | Both | Cross-sectional | QUA | **Fast food:** Yes | 616 | Maternal level of education, husband/partner occupation, fruit/vegetable intake, mode of transport, fast food intake, household wealth status, watching television, type of school, missing meal, physical activity, age | 7 |
| Mihrshahi (2017)[35] | Australia | 5-16y | Both | Cross-sectional | QUA | **Fast food:** ≥1 times/week | 7568 | Age, sex, SES tertile, residential location, cultural background, meeting daily physical activity recommendations (60 mins daily) | 7 |
| Muckelbauer (2016)[36] | Germany | 8.3 (mean age) | Both | Longitudinal study (FU) | 24h recall QUA | **SSB:** Increase by 1 glass/day (1 glass=200 ml) | 1987 | Baseline BMI, baseline consumption of all beverage categories, change in milk, tea and other beverages consumption, age, sex, migrational background, study arm, follow-up duration | 9 |
| Nasreddine (2014)[37] | Lebanon | 6-19y (age-divided) | Both | Cross-sectional | 24-h recall | **Bread/cereals, milk/dairy, meat, fast food, sugar/sweets, SSB:** High consumption (3rd tertile based on percent contribution to daily energy intake) | 868 | Baseline socio-demographic, lifestyle, dietary characteristics | 7 |
| Nguyen (2021)[38] | Vietnam | 8y | Both | Longitudinal study (FU) | FFQ | **Milk/milk products, packaged sweets/snacks:** Highest (4th) quartile | 1961 | Sex, site type, wealth index, interaction term of wealth and site type | 6 |
| Nicklas (2003)[39] | US | 10y | Both | Cross-sectional | 24-h recall | **Vegetables:** + 161 g/day. **Grain:** + 187 g/day. **Meat:** +60 g/day. **Candy:** +40 g/day. SSB: +399 g/day. **Salty snacks:** +12 g/day. **Milk:** +409 g/day. **Cheese:** 22 g/day. | 1562 | Total calorie intake, age, study year, ethnicity, gender, and ethnicity | |
| Notara (2020)[40] | Greece | 10-12y | Both | Cross-sectional | FFQ | **Dietary fibers:** >15 g/1000 kcal/day | 1659 | Age, gender, breakfast consumption, daily walking time, computer use, parental education level, parental BMI, KIDMED index | 7 |
| O´Niel (2011)[41] | US | 2-18y | Both | Cross-sectional | 24-h dietary recall interview | **Chocolate candy, sugar candy:** Consumers | 11181 | Gender, ethnicity, age, energy | 9 |

| | | | | | | | | | |
|---|---|---|---|---|---|---|---|---|---|
| Payab (2015)[42] | Iran | 6-18y | Both | Cross-sectional | FFQ | **Sweets, SSB, fast food, salty snacks:** Daily | 13486 | Family history of chronic disease, physical activity, screen time, socio-economic status. | 7 |
| Pengpid (2016)[43] | Cambodia, Indonesia, Malaysia, Myanmar, Thailand, the Philippines, Vietnam | 13-15y | Both (separate) | Cross-sectional | FFQ - The Global School based Student Health Survey | **Fast food:** ≥2 times/week. **Fruits**: ≥2 servings/day. **Vegetables:** ≥3 servings/day. | 2261 | Age, country income, diet, hunger, tobacco use, active transport, sedentary behavior, psychosocial and social-familial factors | 5 |
| Pirincci (2010)[44] | Turkey | 6-11y | Both | Cross-sectional | QUA | **Fast food:** ≥2 times/week | 3642 | Variables with significant associations (i.e. p-value <0.05) in the bivariate logistic regressions | 6 |
| Sakaki (2019)[45] | US | 9-16y | Both (separate) | Cross-sectional | FFQ | **100% OJ consumption:** >1 glass/day | 1308 | Cohort, age, race, total energy intake excluding OJ, moderate/physical activity, screen time | 8 |
| Sanigorski (2007)[46] | Australia | 4-12y | Both | Cross-sectional | FFQ | **Fruit, vegetables, fruit juice/drinks, soft drinks:** ≥2/day. **Fast food:** >1/week. | 1944 | Age, gender, socio-economic status | 7 |
| Santiago (2013)[47] | Spain | 6-12y | Both (separate) | Cross-sectional | FFQ | **Fruit:** ≥2/day. **Buns, sweets:** ≥1/day. **Fast food:** ≥1/week. | 2814 | Sport activities, breakfast consumption, dietary intake (fruit, buns, fast food, sweet) | 7 |
| Shan (2010)[48] | China | 6-18y | Both | Cross-sectional | QUA | **SSB, fast food:** ≥3 times/week. | 21198 | Age, gender, Tanner stage, urban/rural residence | 7 |
| Shin (2017)[49] | Korea | 6-18y | Both | Cross-sectional | The Student Health Examination and Survey | **Cereal (instant noodles), SSB, fast food, milk, meat:** Every day. | 3225 | Gender, survey year, school grade, food intakes/week and breakfast | 7 |
| Siddarth (2013)[50] | US | 7-18y (age-divided) | Both | Cross-sectional | FFQ | **Fast food:** ≥3 meals/week | 1956 | Physical and sedentary activity level, sex, ethnicity, income level | 8 |
| Valente (2011)[51] | Portugal | 5-10y | Both (separate) | Cross-sectional | FFQ | **SSB:** ≥3 servings/day | 1675 | Energy intake, parents' education level, time of sleep, questionnaire responder, total carbohydrates, sugars, MUFA, television watching | 7 |
| Vinciguerra (2019)[52] | Italy | 6-15y | Both | Cross-sectional | FFQ (HBSC) | **SSB:** Drinkers | 1702 | Gender, level of PA ST, SSB, parental risk factors | 7 |
| Walsh (2020)[53] | US | 2-12y | Both | Cross-sectional | The Beverage and Snack QUA - total monthly consumption | **Salty snacks, SSB, Sweet snacks:** Each additional monthly consumption | 300 | Child age, child sex, race, caregiver education, NFS household income, FV access, food insecurity | 7 |

| | | | | | | | | | |
|---|---|---|---|---|---|---|---|---|---|
| White (2020)[54] | US | 2-20y | Both | Cross-sectional | Diet behavior and nutrition interview (NHANES) | **Milk:** Daily | 20039 | Age, race/ethnicity, daily milk consumption, income, NHANES cycle. | 7 |
| Wijnhoven (2015)[55] | Bulgaria, Lithuania, Portugal, Sweeden, Czech Republic | 6-9y | Both | Cross-sectional | FFQ (HBSC) | **Fruit, vegetables:** ≥7 days/week. **SSB, Salty snacks, Sweets, Sweet bakery (cakes), Fast food:** ≥3 days/week. | 8512 | Children's sex, age, all thirteen health-risk behaviors, children's residential urbanization grade, parental education, parental occupation, random effects for the primary sampling units | 7 |
| Xu (2016)[56] | China | 7-12y | Both | Cross-sectional | QUA | **SSB:** ≥3/week | 4644 | Not specified | 5 |
| Xue (2016)[57] | China | 6-18y | Both (separate) | Longitudinal study (BA - Wave 2004) | China Health and Nutritional Survey | **Fast food:** ≥1 time in past 3 months | 1497 | Age, ethnicity, household income, urbanicity, geographical region and physical activity levels | 8 |
| Zhang (2016)[58] | China | 7-12y | Both | Cross-sectional | FFQ (past 7 days) | **Fruits, Vegetables, Meat:** + servings/day. **SSB:** + cups/day. **Fried food, Western fast food:** + times/week. | 3766 | Age, gender, only child or not, paternal and maternal educational level, paternal and maternal occupation, monthly household income | 7 |
| Zhang (2018)[59] | China | 6-10y | Both | Cross-sectional | FFQ | **SSB:** ≥1 times/day. **Vegetables:** ≥1 times/day. | 13001 | Age, sex, sleep, outdoor activity, vegetables intake, snack intake, SSB intake | 5 |
| Zhao (2017)[60] | China | 6-16y | Both | Longitudinal study (BA) | FFQ (past 3 months) | **Fast food:** ≥3 times/week | 1626 | Child factors (age, sex, and school location) and maternal factors (BMI and education level) | 7 |

Characteristics of included trials. Abbreviations: NOS = Newcastle-Ottawa Scale (risk of bias and study quality assessment score); FFQ = Food Frequency Questionnaire; YAQ = Youth and Adolescents Questionnaire; SSB = Sugar-sweetened beverages; QUA = Questionnaire; HBSC = Health Behavior in School-aged Children; FU = follow-up data used for meta-analysis; BA = baseline data used for meta-analysis.

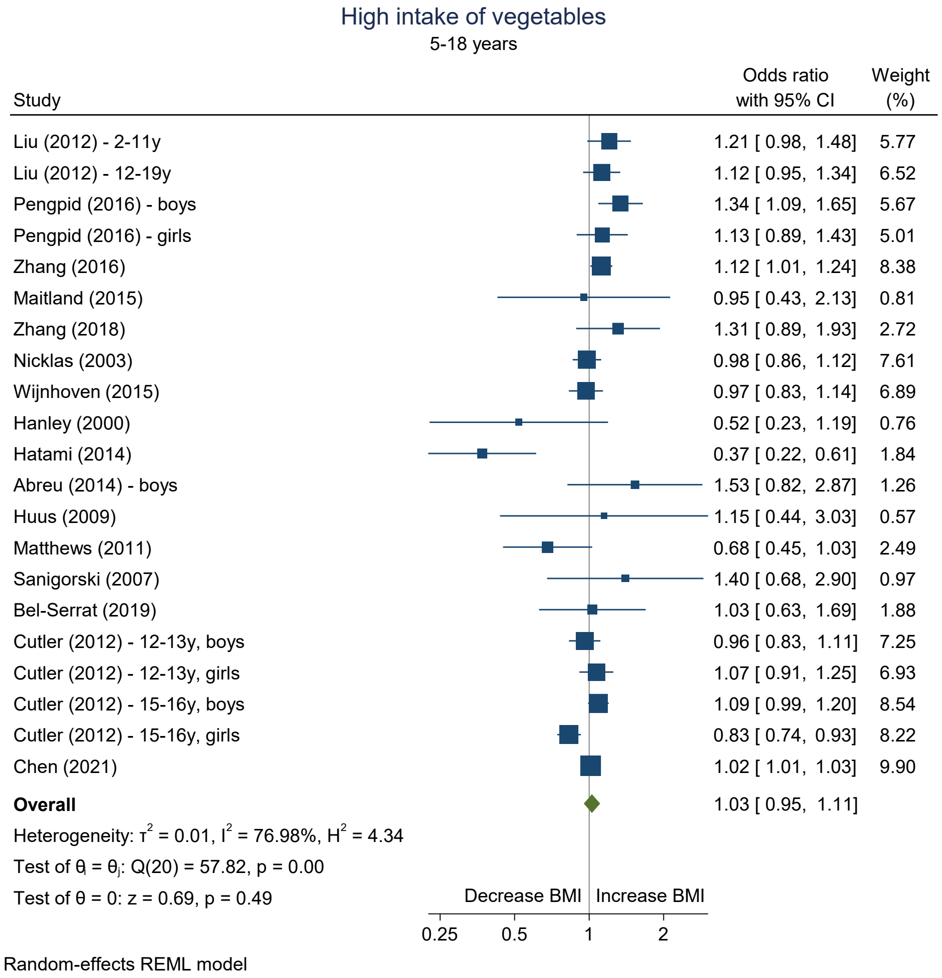


**Figure S1.** Association between a high intake of vegetables and risk of overweight/obesity in children and adolescents 5-18 years.

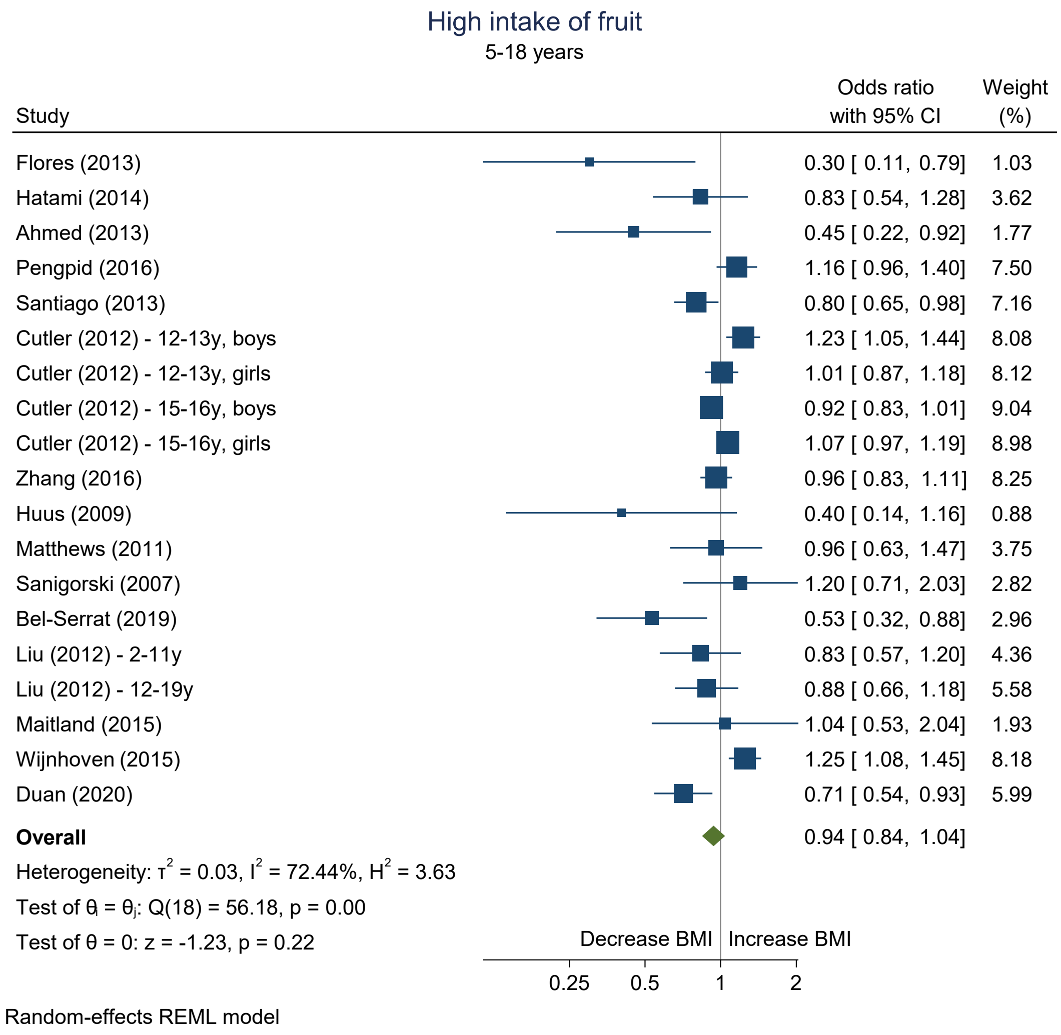


**Figure S2.** Association between a high intake of fruit and risk of overweight/obesity in children and adolescents 5-18 years.

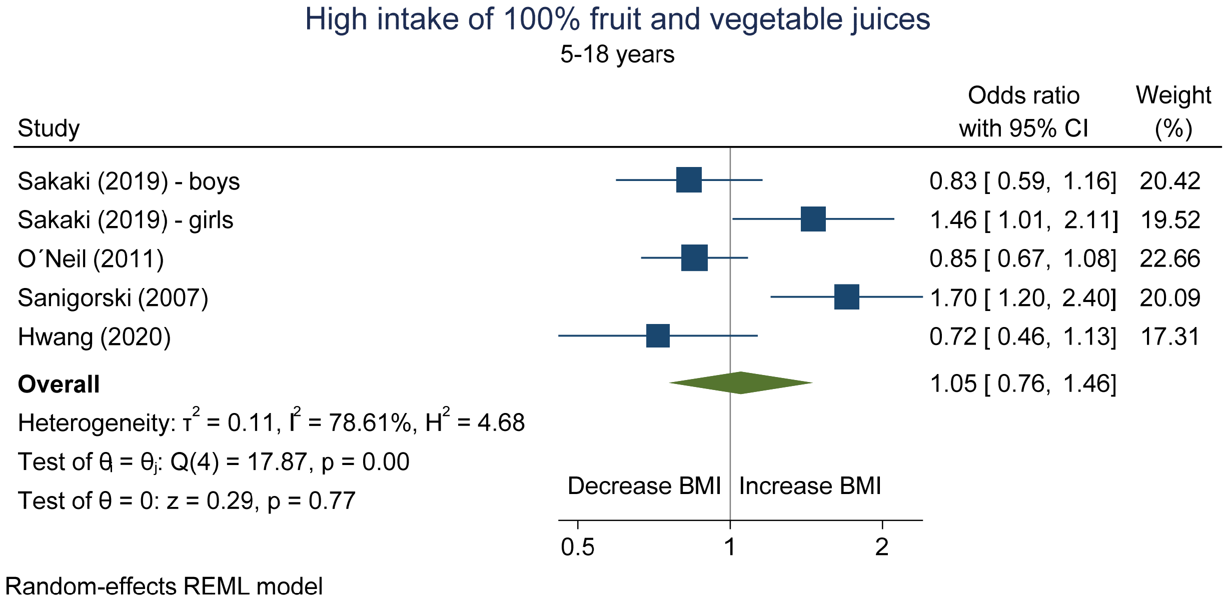


**Figure S3.** Association between a high intake of 100% fruit and vegetable juices and risk of overweight/obesity in children and adolescents 5-18 years.

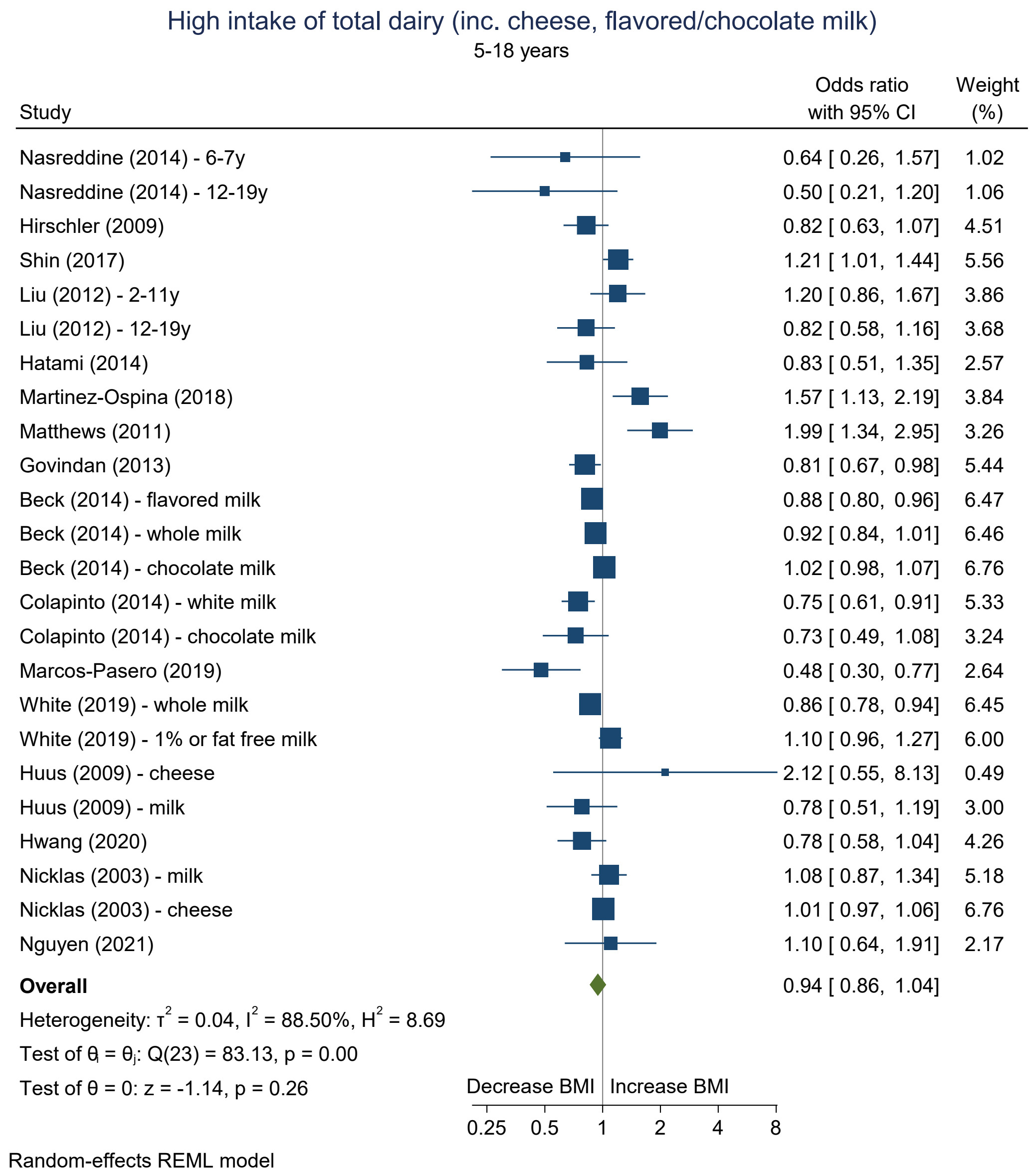


**Figure S4.** Association between a high intake of total dairy and risk of overweight/obesity in children and adolescents 5-18 years.

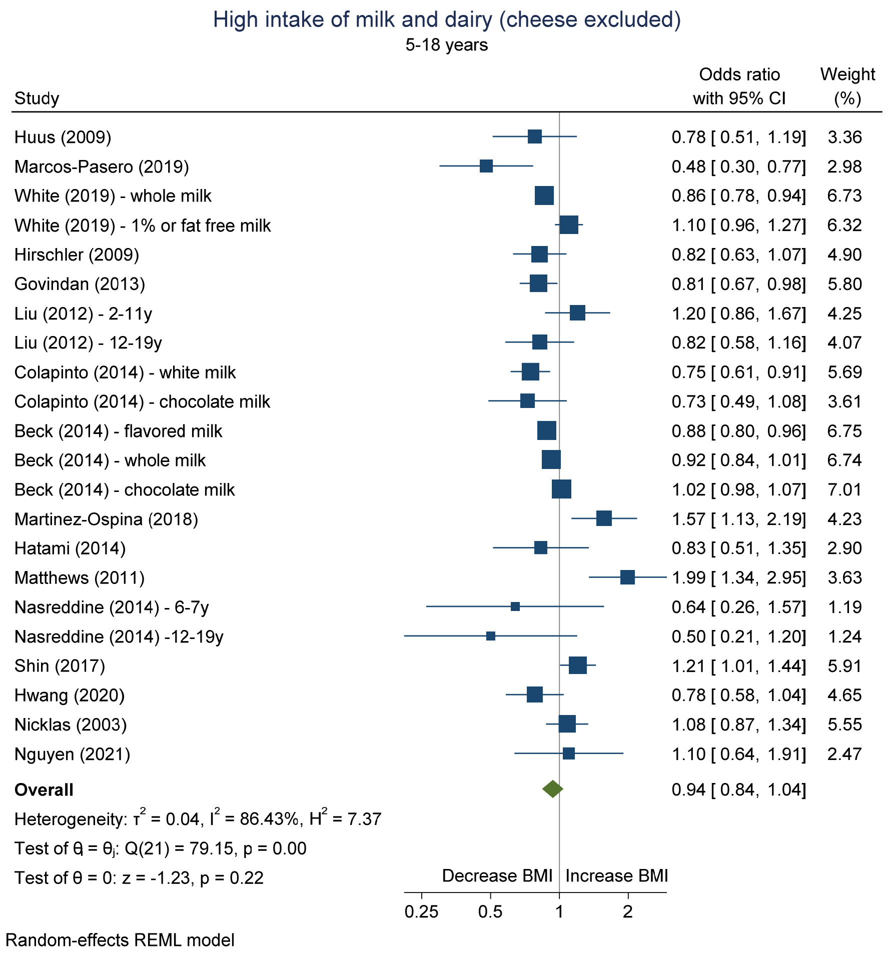


**Figure S5.** Association between a high intake of milk and dairy and risk of overweight/obesity in children and adolescents 5-18 years.

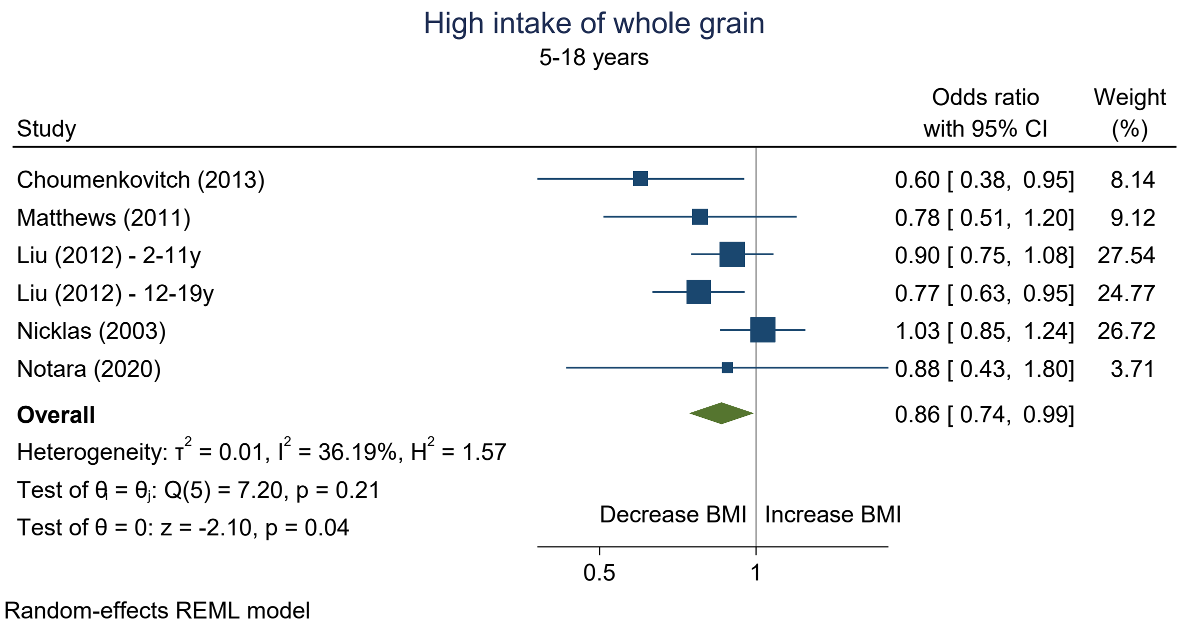


**Figure S6.** Association between a high intake of whole grain and risk of overweight/obesity in children and adolescents 5-18 years.

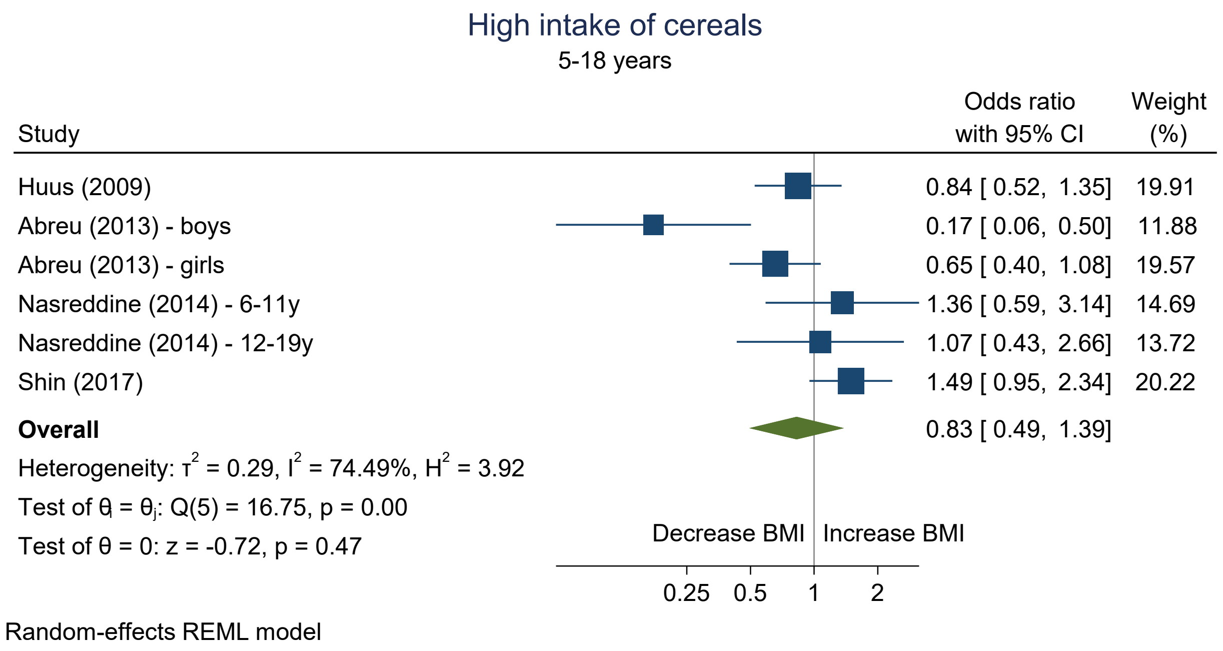


**Figure S7.** Association between a high intake of cereals and risk of overweight/obesity in children and adolescents 5-18 years.

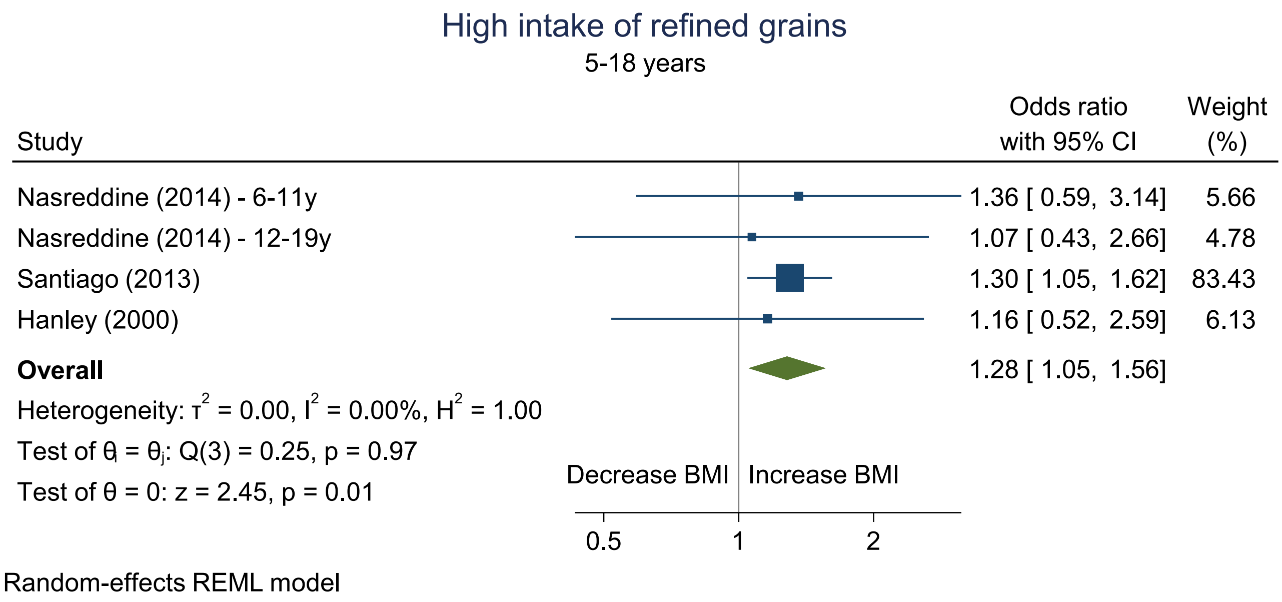


**Figure S8.** Association between a high intake of refined grains and risk of overweight/obesity in children and adolescents 5-18 years.

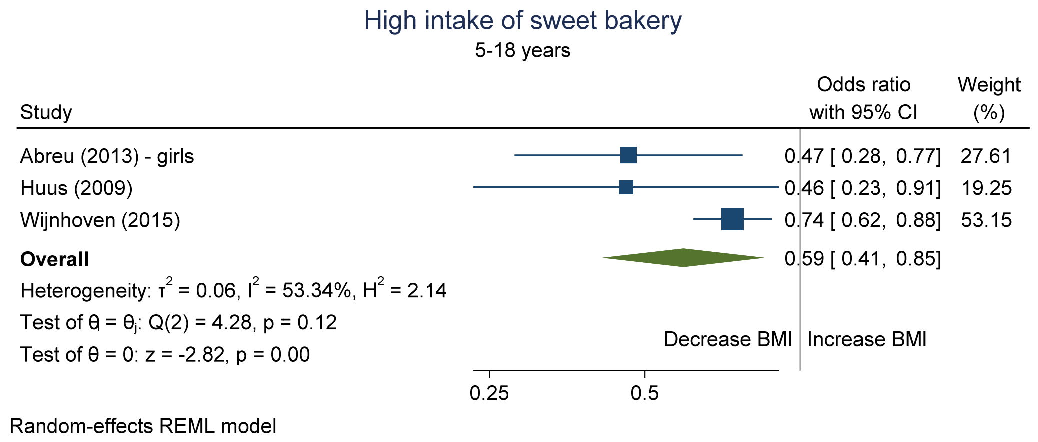


**Figure S9.** Association between a high intake of sweet bakery and risk of overweight/obesity in children and adolescents 5-18 years.

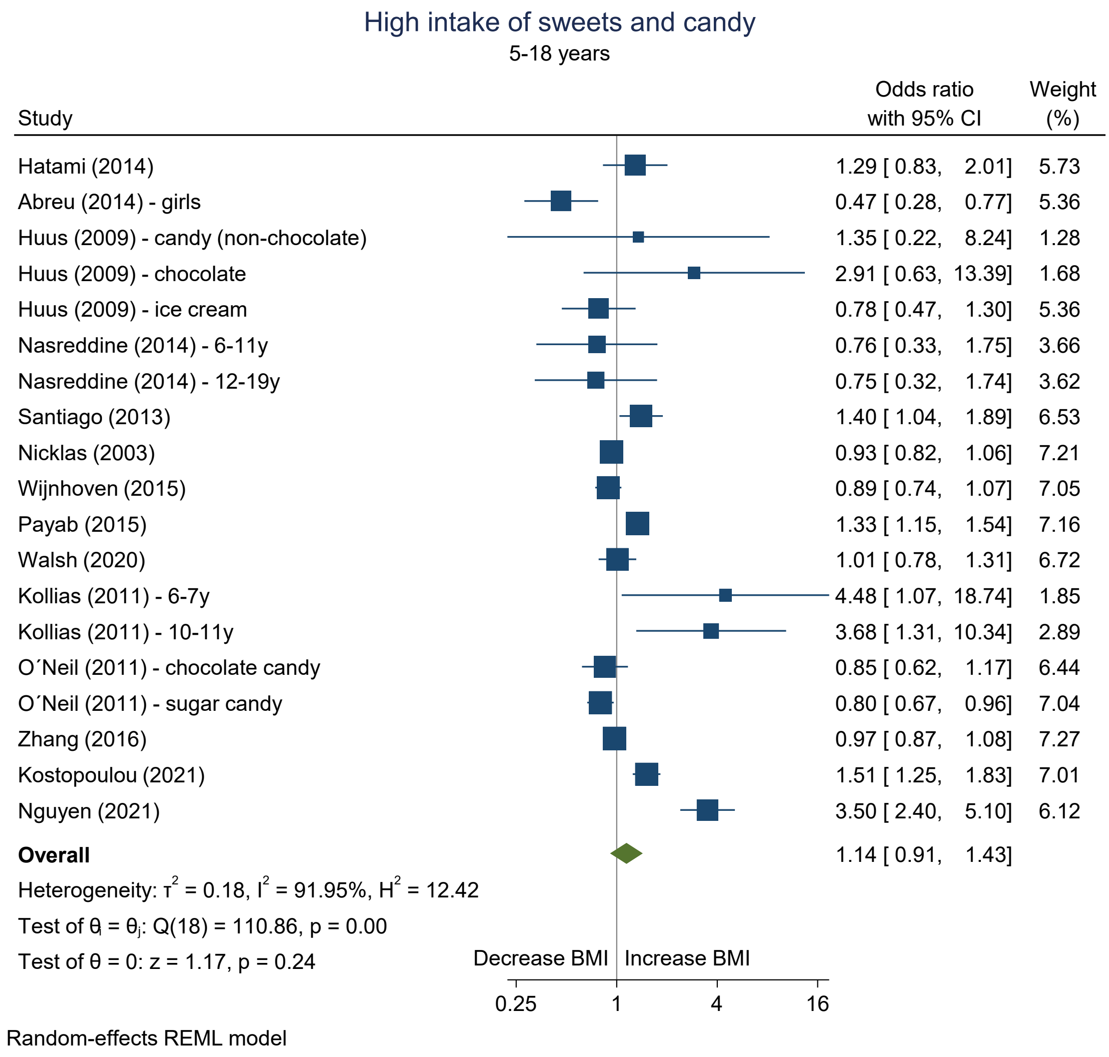


**Figure S10.** Association between a high intake of sweets and candy and risk of overweight/obesity in children and adolescents 5-18 years.

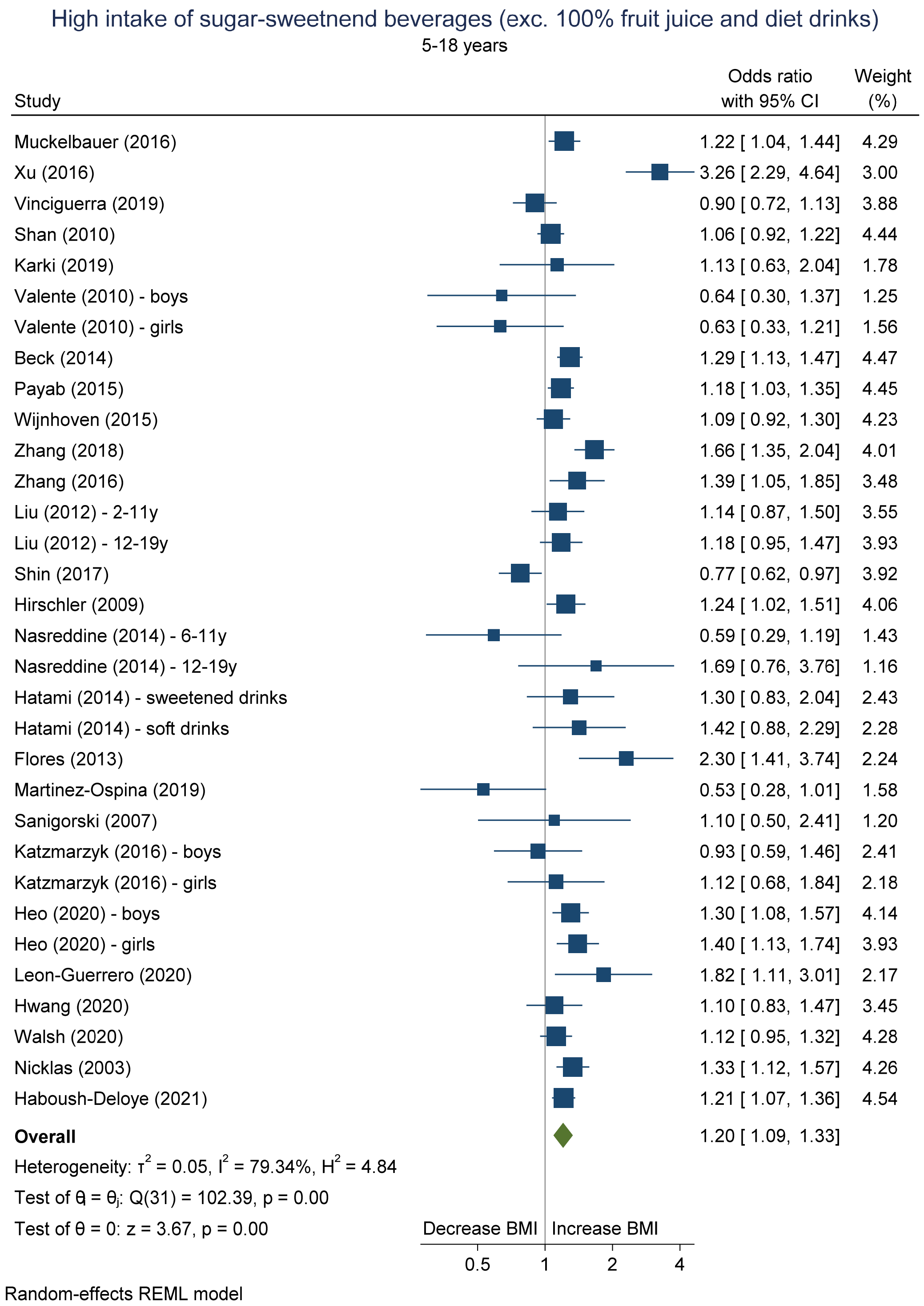


**Figure S11.** Association between a high intake of sugar-sweetened beverages and risk of overweight/obesity in children and adolescents 5-18 years.

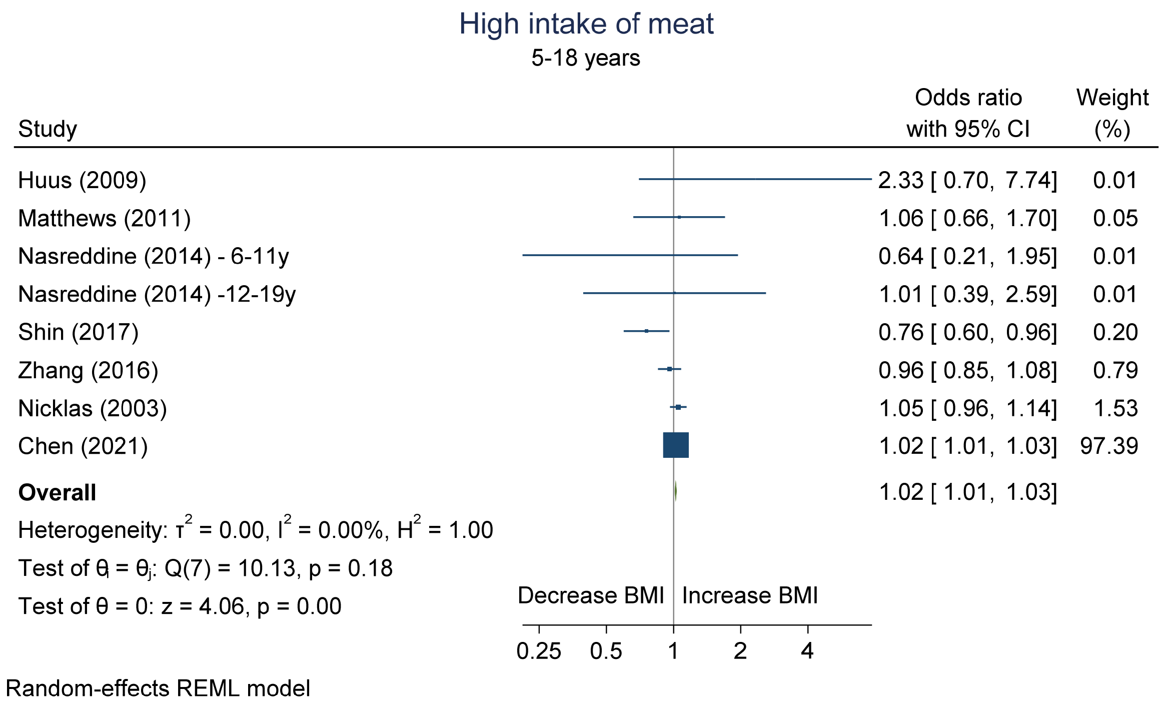


**Figure S12.** Association between a high intake of meat and risk of overweight/obesity in children and adolescents 5-18 years.

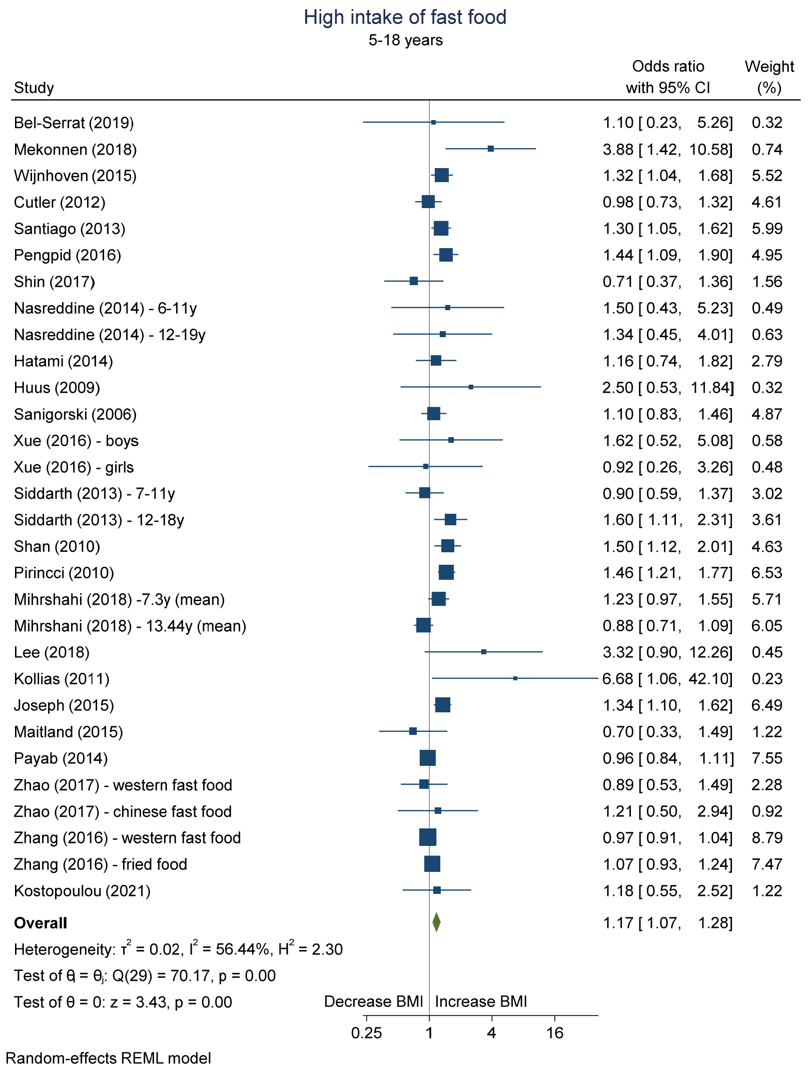


**Figure S13.** Association between a high intake of fast food and risk of overweight/obesity in children and adolescents 5-18 years.

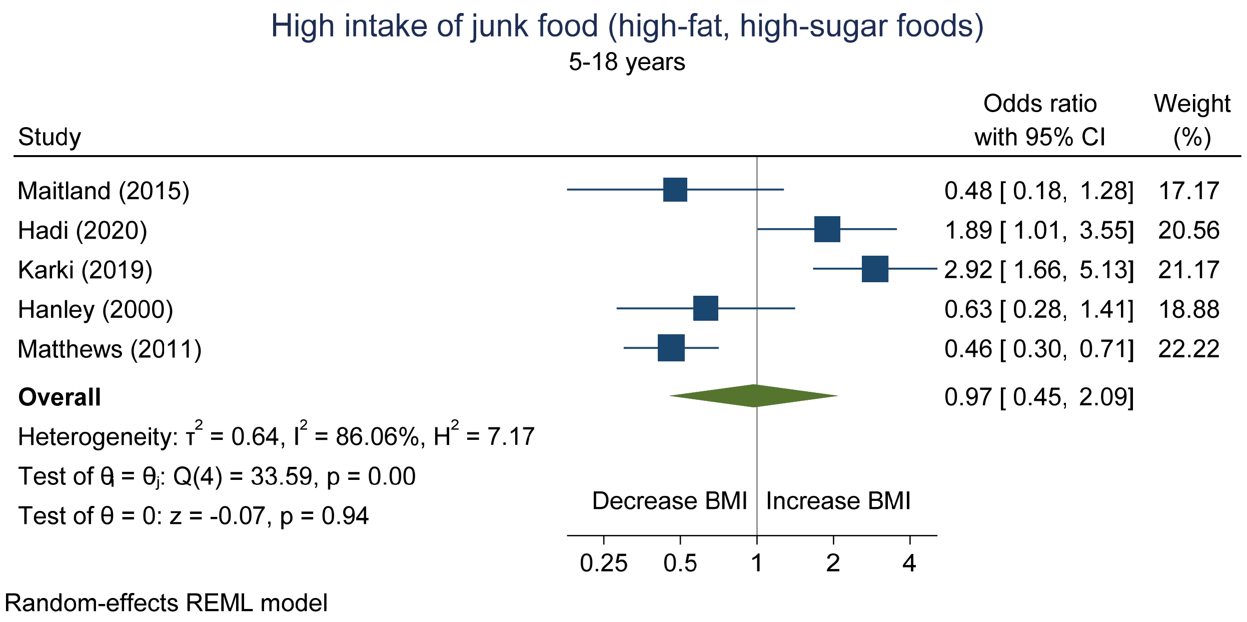


**Figure S14.** Association between a high intake of junk food and risk of overweight/obesity in children and adolescents 5-18 years.

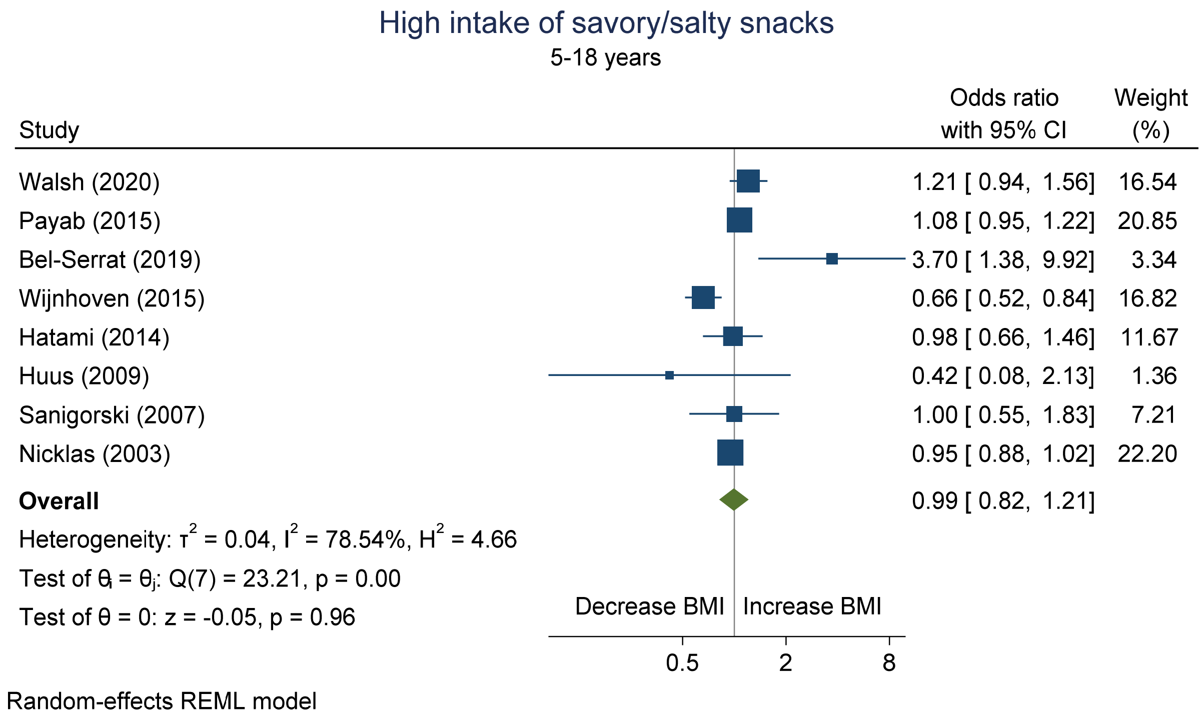


**Figure S15.** Association between a high intake of savory/salty snacks and risk of overweight/obesity in children and adolescents 5-18 years.

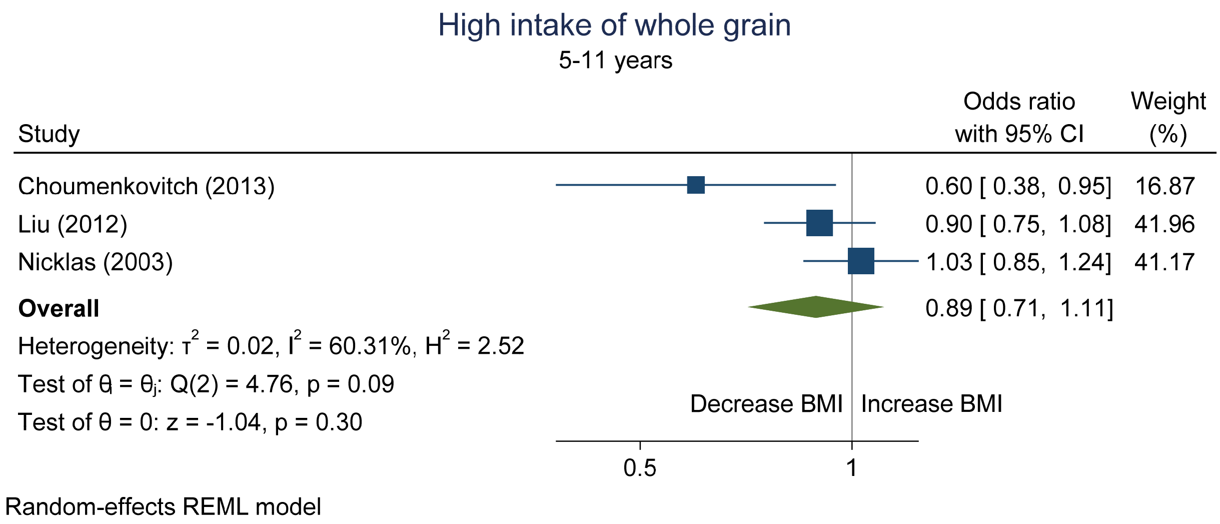


**Figure S16.** Association between a high intake of whole grain and risk of overweight/obesity in children and adolescents 5-11 years.

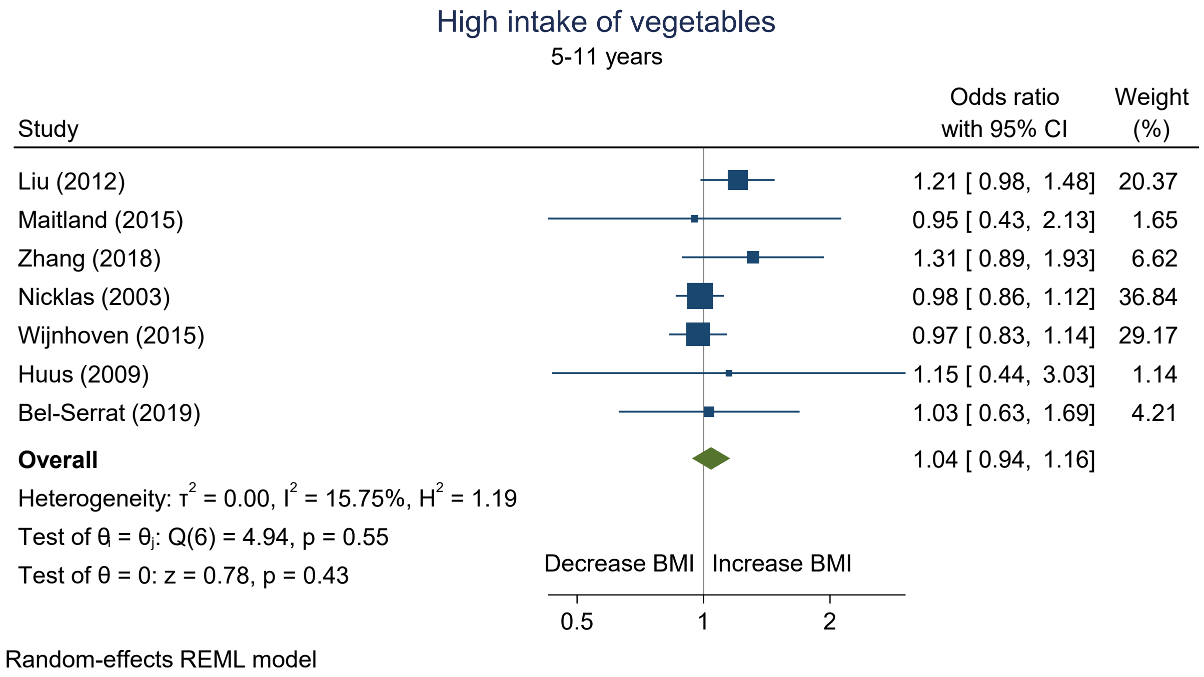


**Figure S17.** Association between a high intake of vegetables and risk of overweight/obesity in children and adolescents 5-11 years.

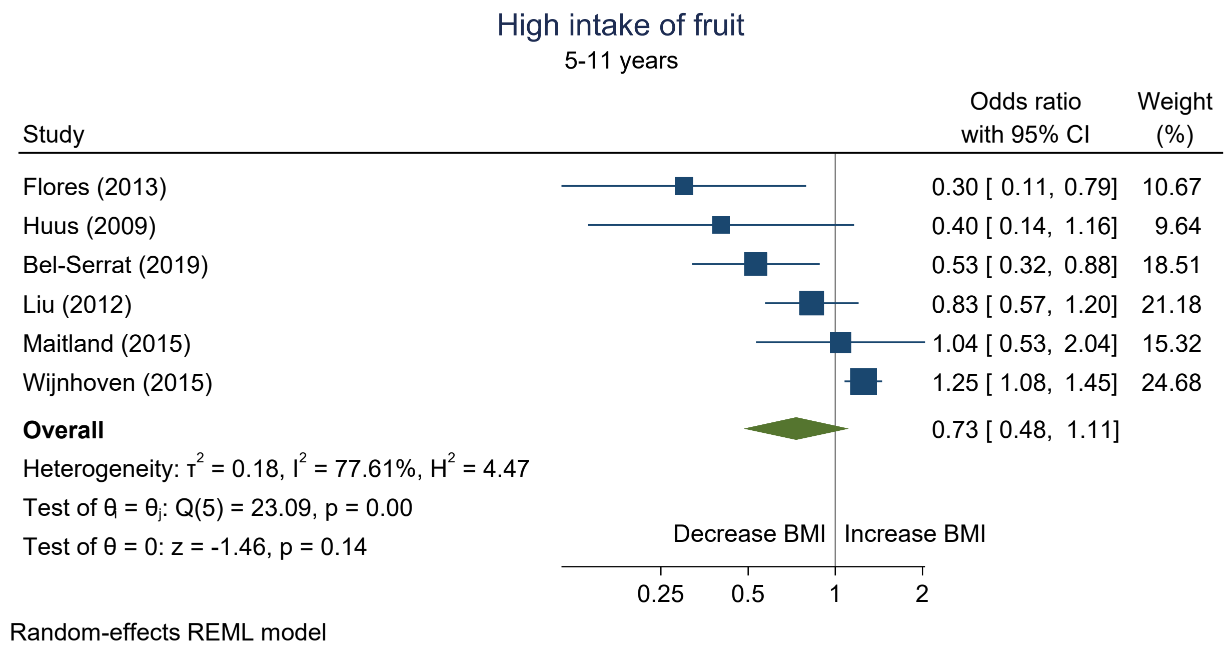


**Figure S18.** Association between a high intake of fruit and risk of overweight/obesity in children and adolescents 5-11 years.

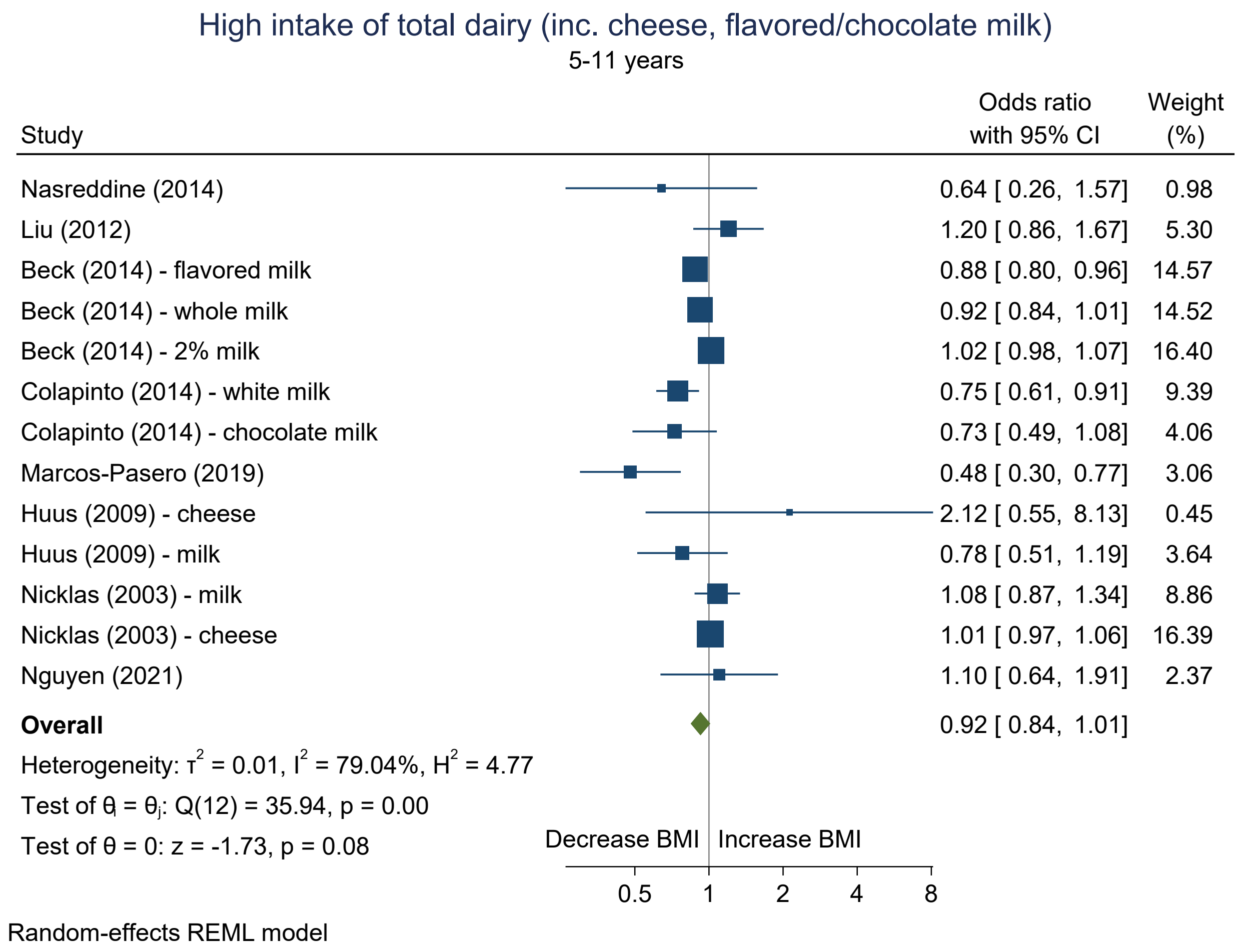


**Figure S19.** Association between a high intake of total dairy and risk of overweight/obesity in children and adolescents 5-11 years.

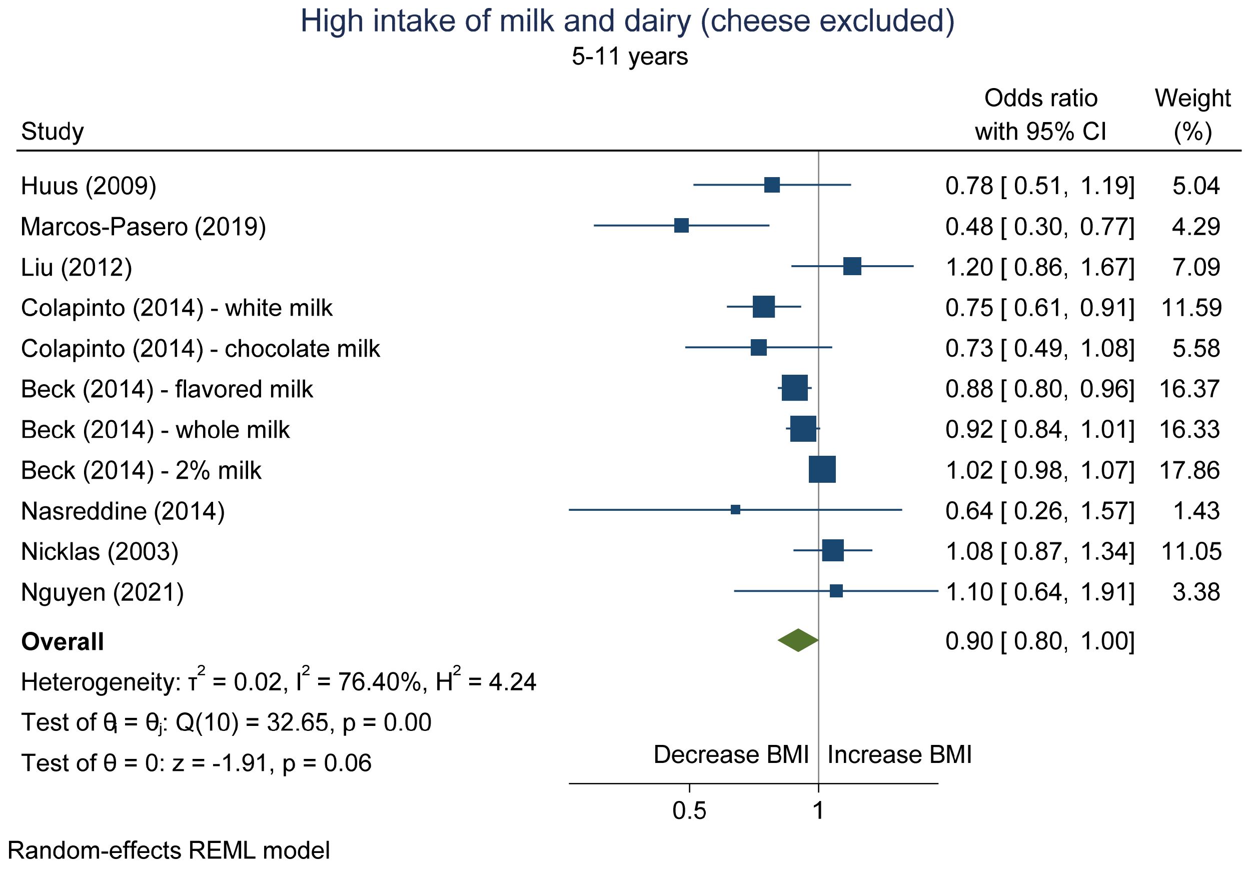


**Figure S20.** Association between a high intake of milk and dairy and risk of overweight/obesity in children and adolescents 5-11 years.

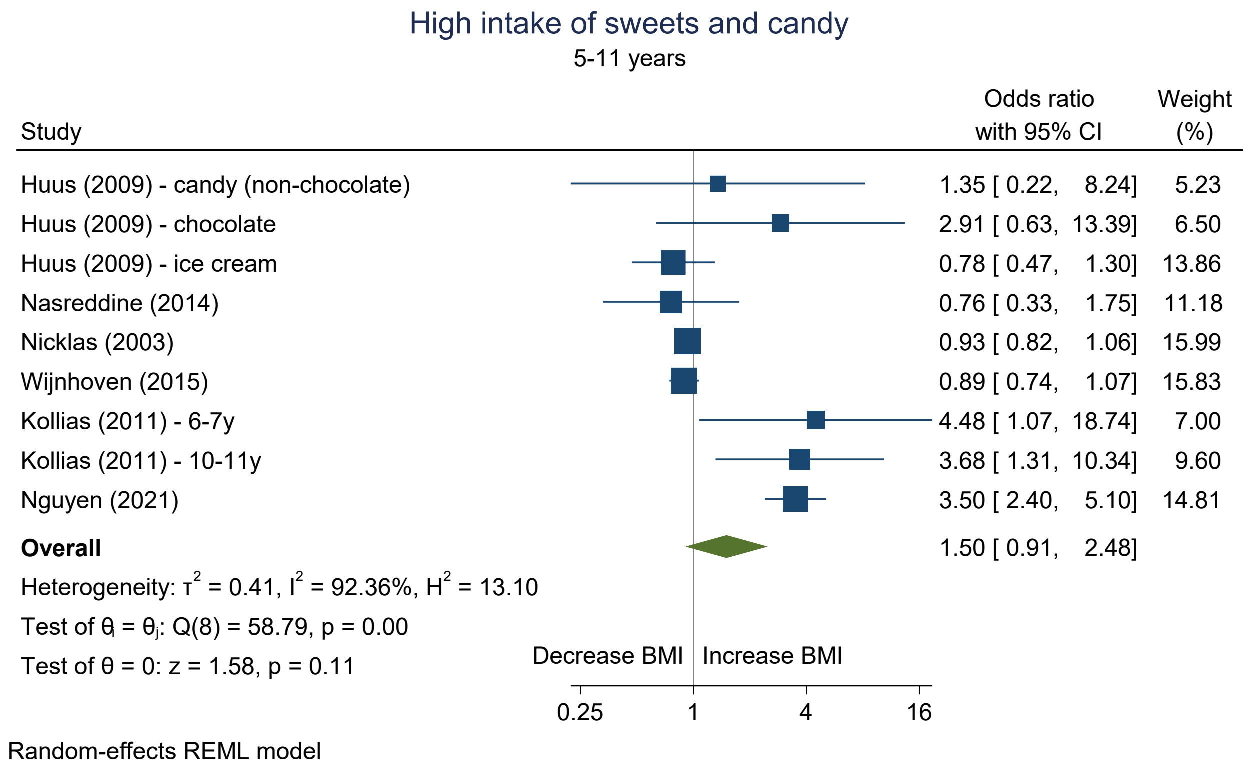


**Figure S21.** Association between a high intake of sweets and candy and risk of overweight/obesity in children and adolescents 5-11 years.

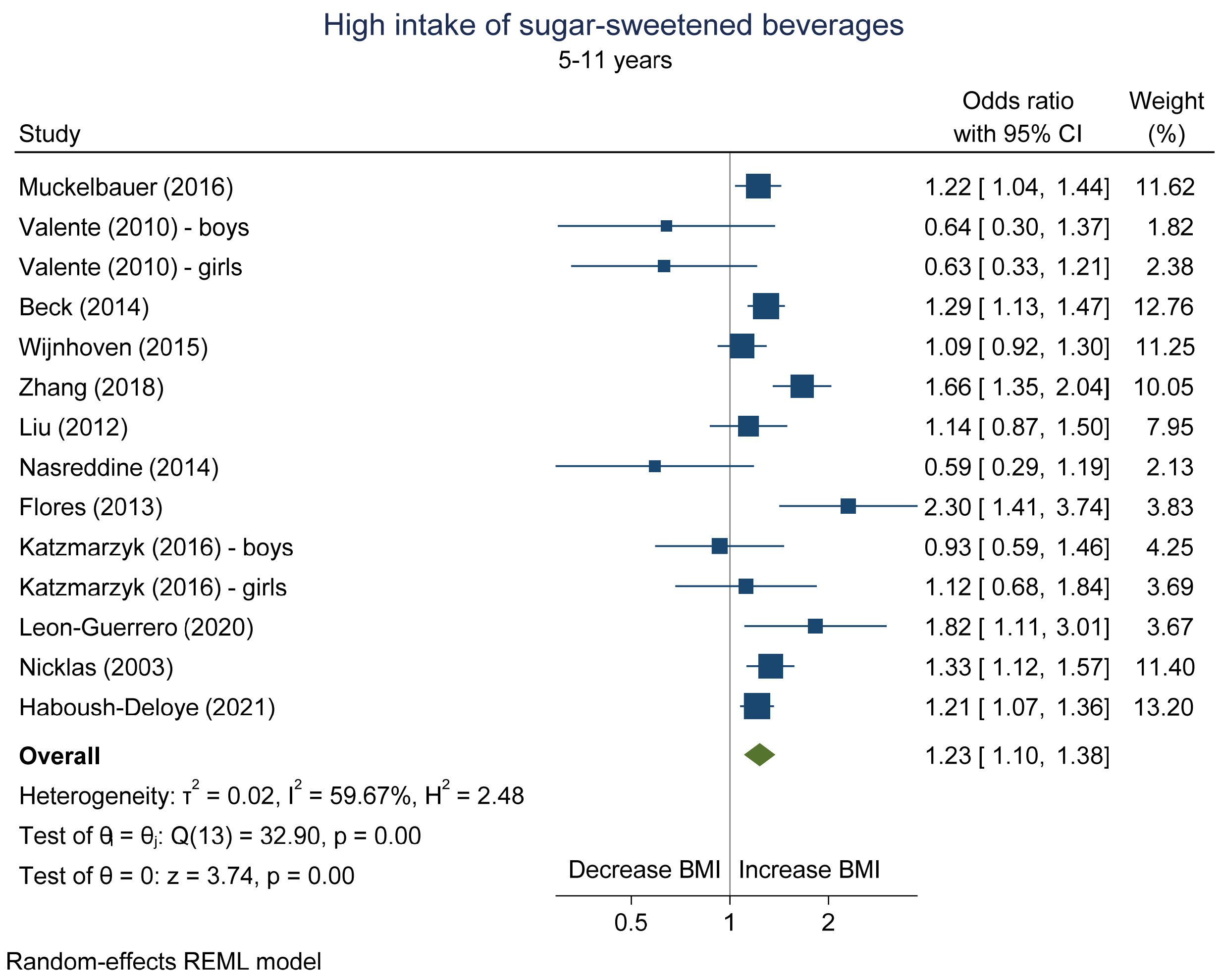


**Figure S22.** Association between a high intake of sugar-sweetened beverages and risk of overweight/obesity in children and adolescents 5-11 years.

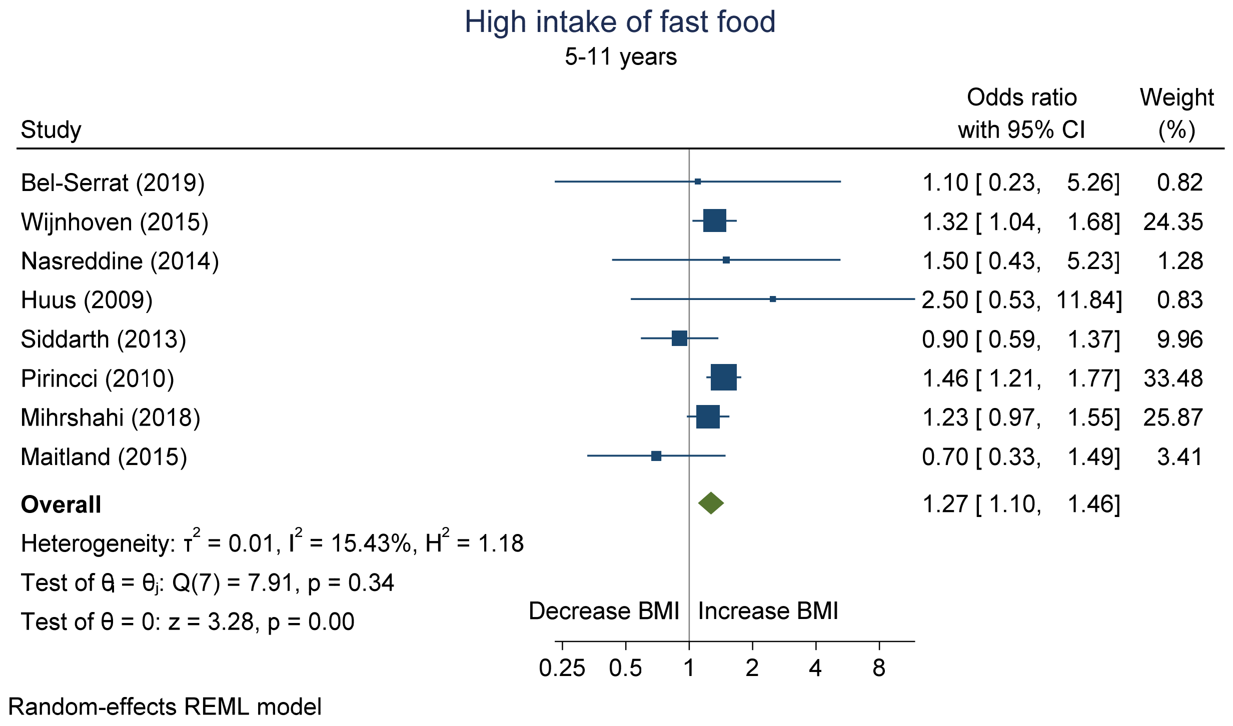


**Figure S23.** Association between a high intake of fast food and risk of overweight/obesity in children and adolescents 5-11 years.

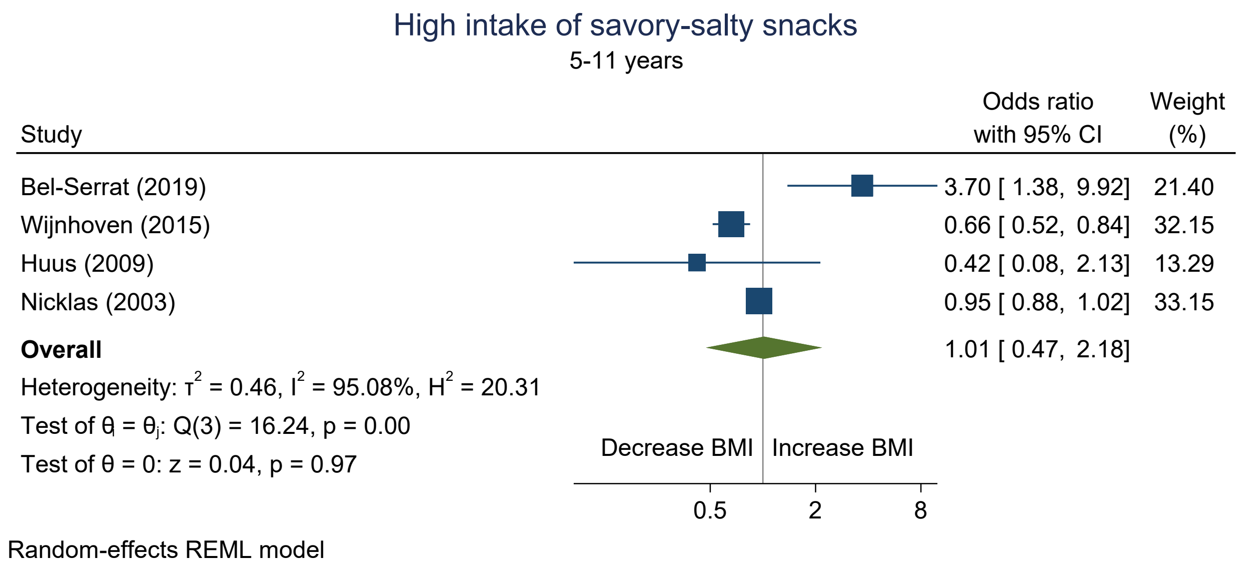


**Figure S24.** Association between a high intake of savory-salty snacks and risk of overweight/obesity in children and adolescents 5-11 years.

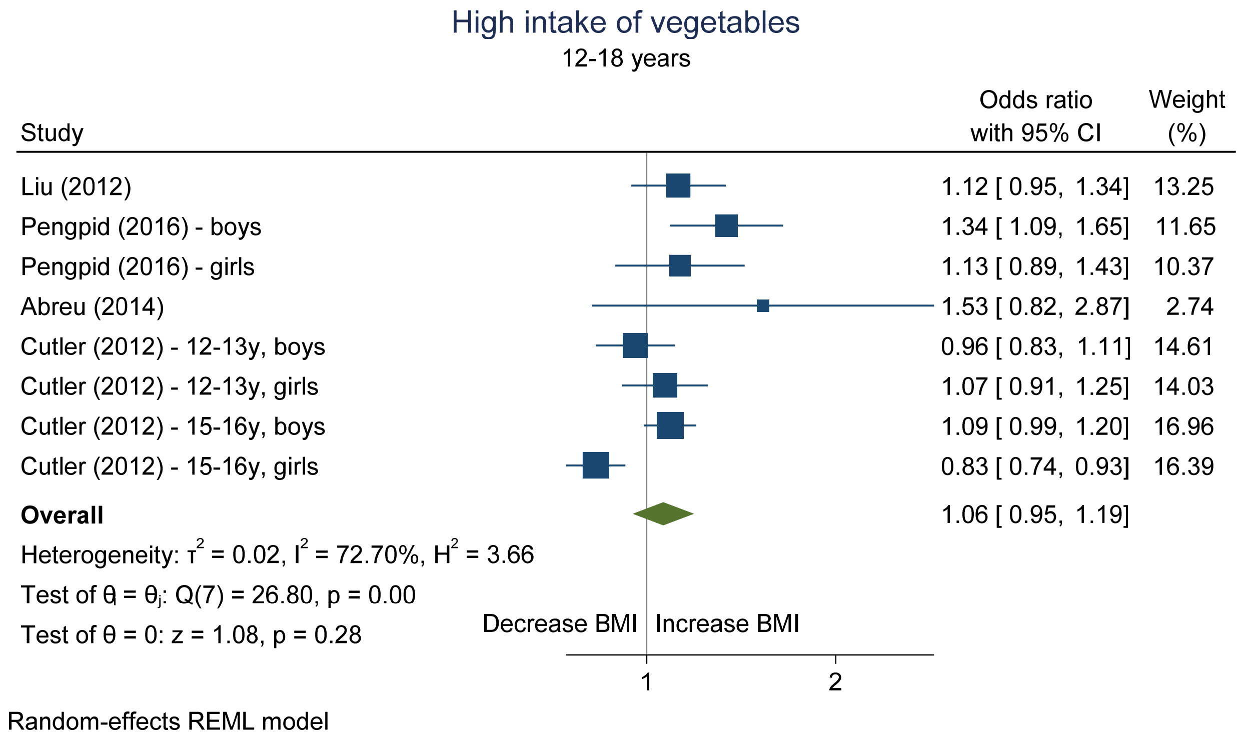


**Figure S25.** Association between a high intake of vegetables and risk of overweight/obesity in children and adolescents 12-18 years.

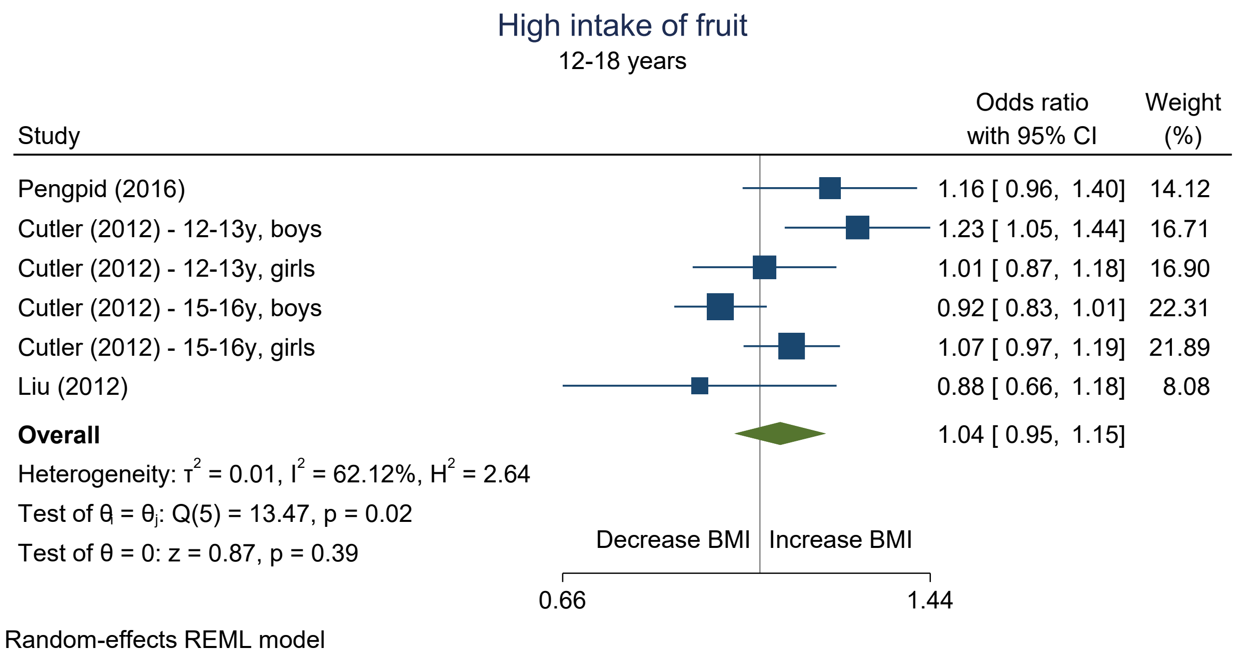


**Figure S26.** Association between a high intake of fruit and risk of overweight/obesity in children and adolescents 12-18 years.

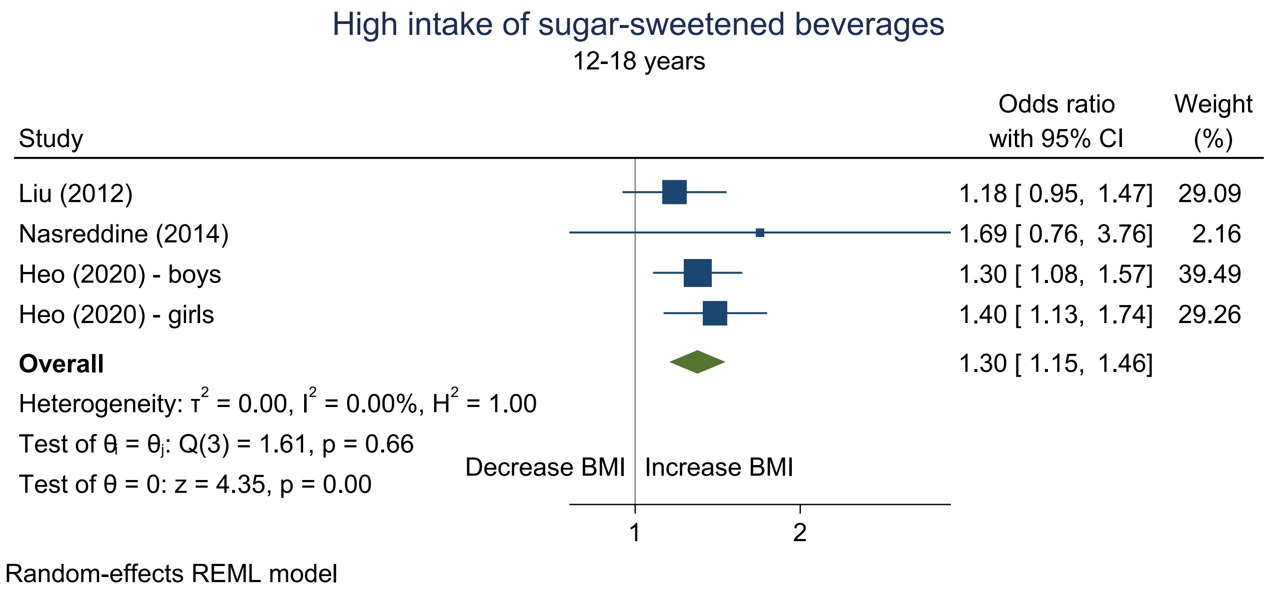


**Figure S27.** Association between a high intake of sugar-sweetened beverages and risk of overweight/obesity in children and adolescents 12-18 years.

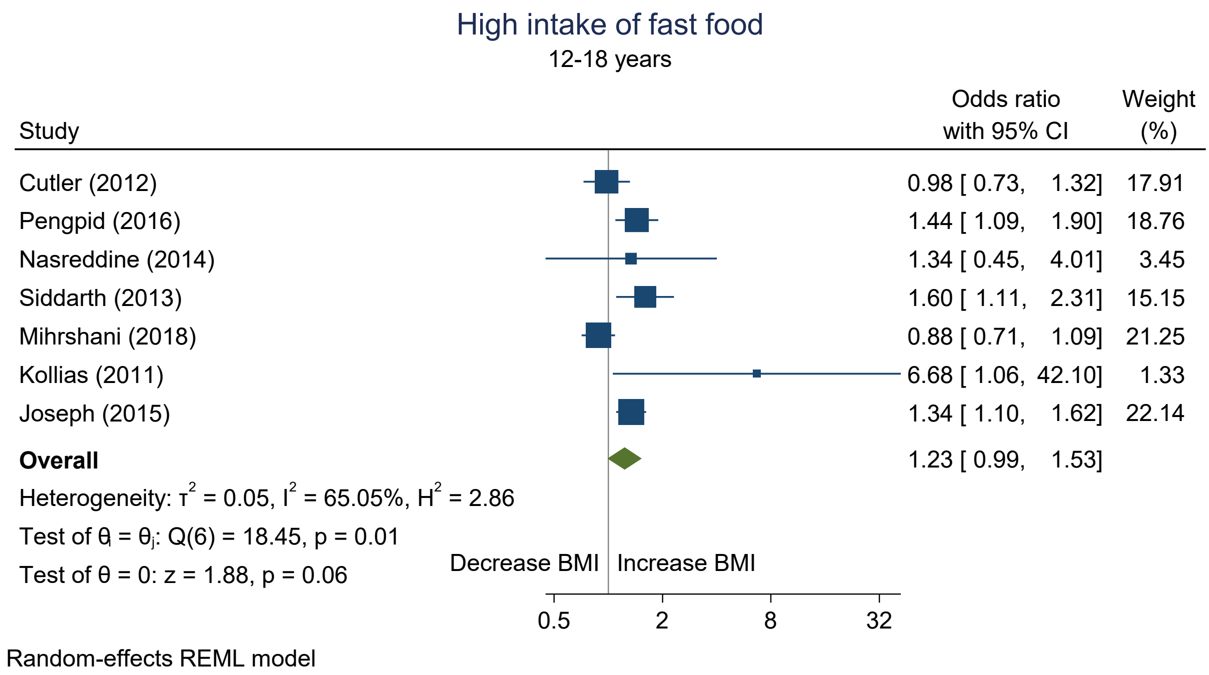


**Figure S28.** Association between a high intake of fast food and risk of overweight/obesity in children and adolescents 12-18 years.

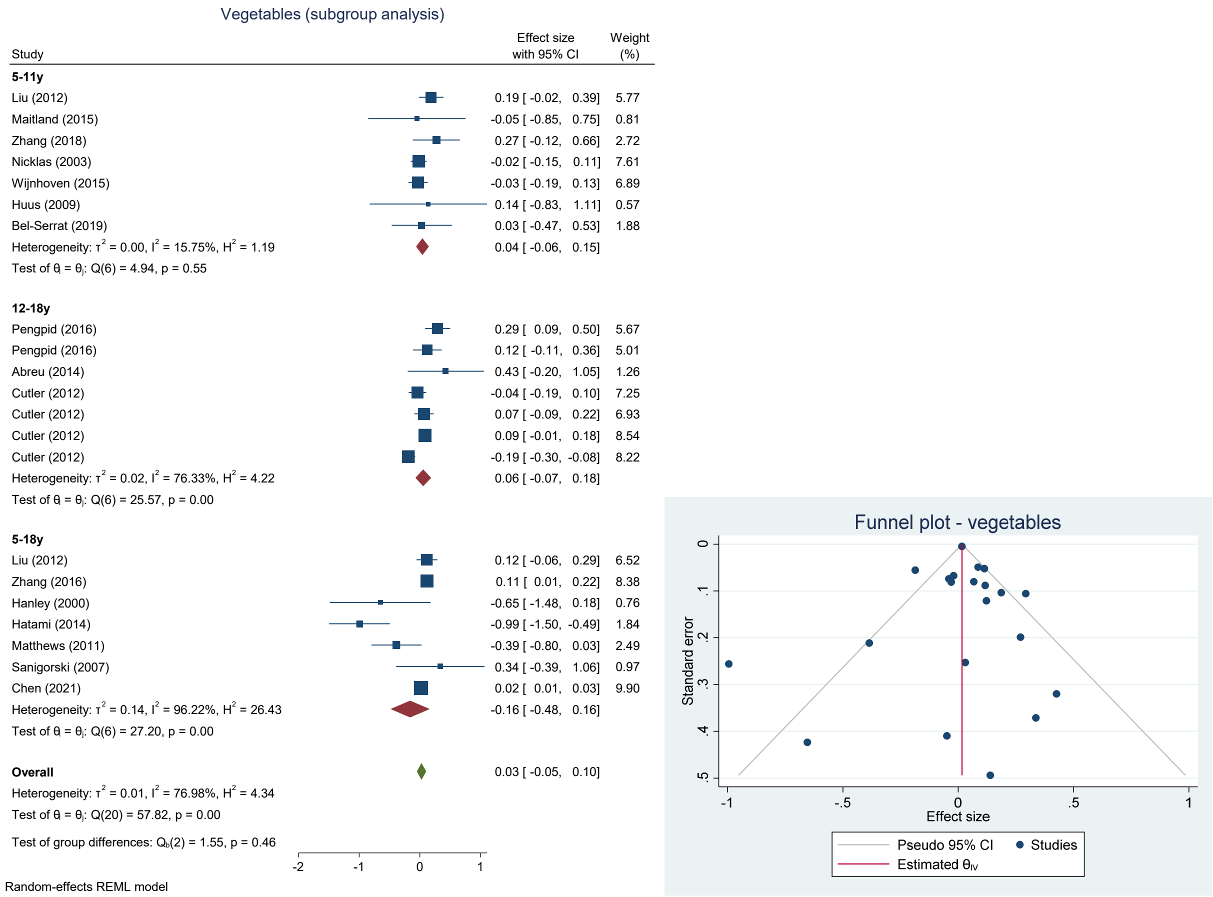


**Figure S29.** Subgroup analysis according to age and funnel plot investigating heterogeneity - vegetables.

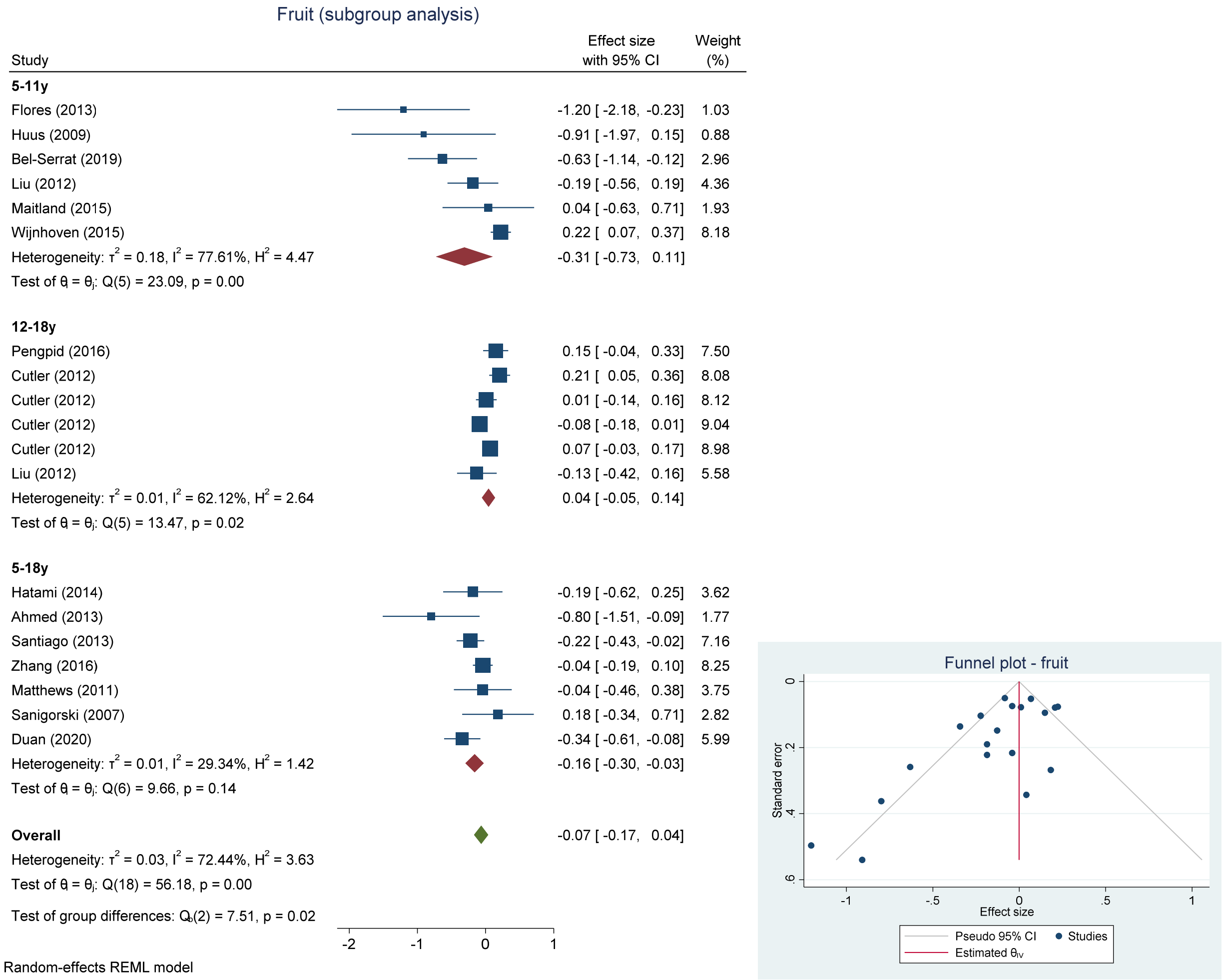


**Figure S30.** Subgroup analysis according to age and funnel plot investigating heterogeneity - fruit.

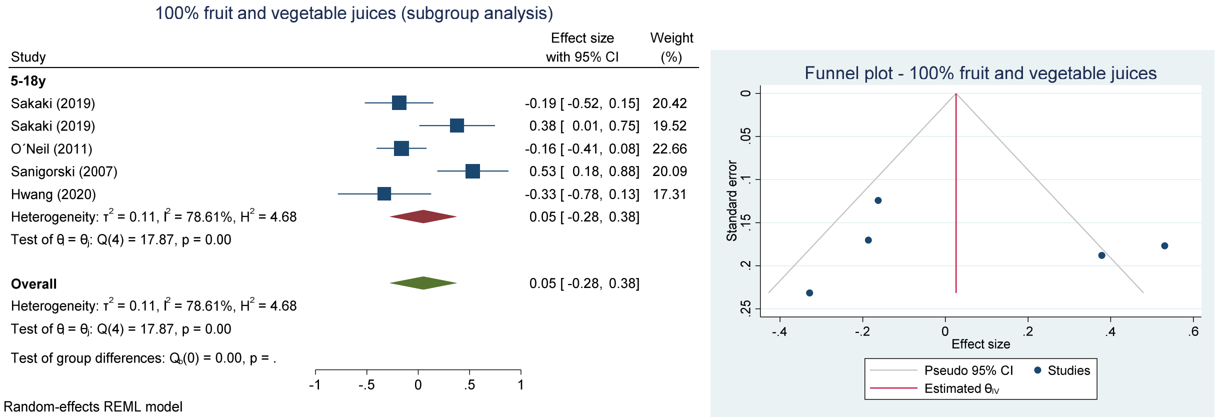


**Figure S31.** Subgroup analysis according to age and funnel plot investigating heterogeneity – 100% fruit and vegetables juices.

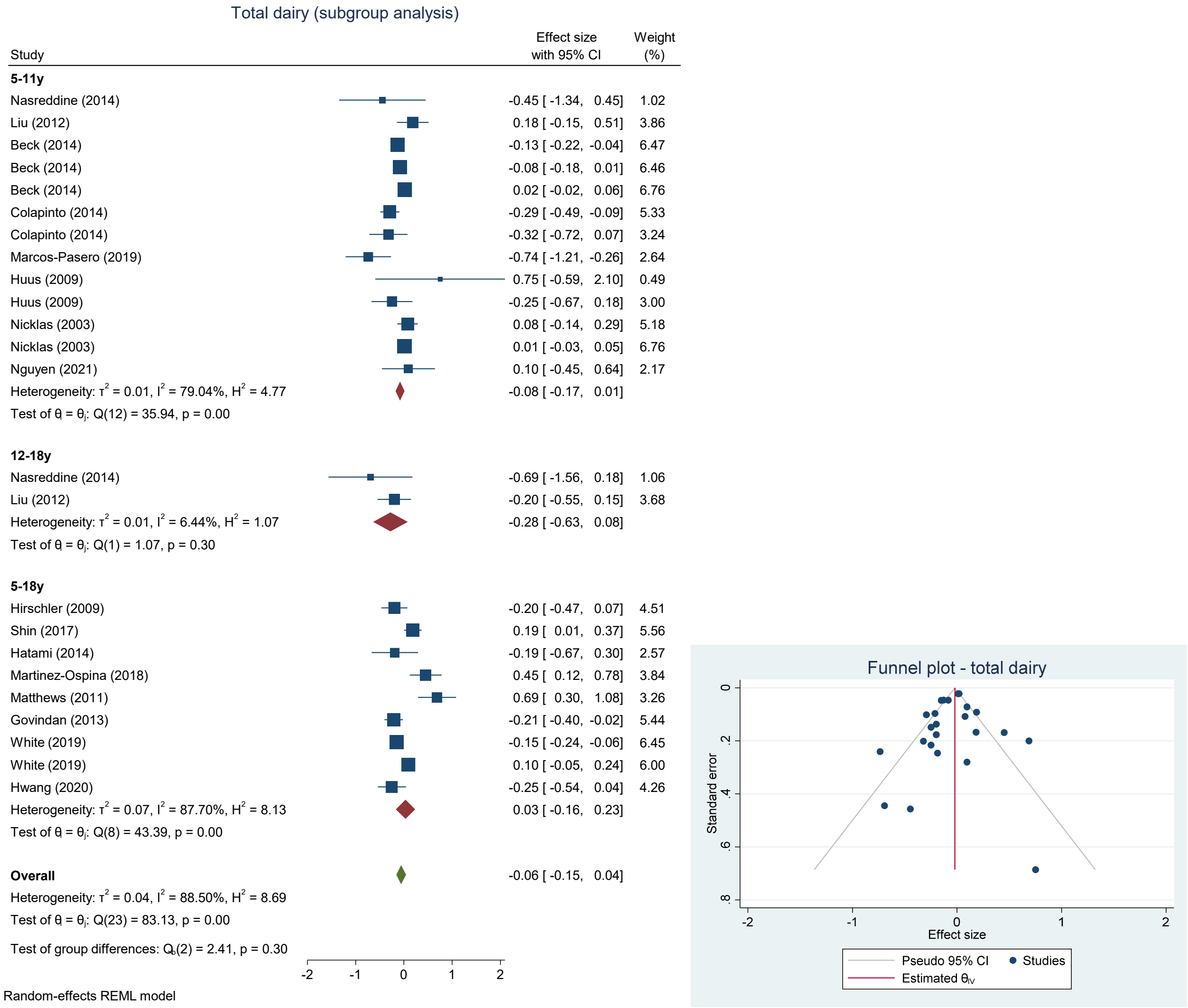


**Figure S32.** Subgroup analysis according to age and funnel plot investigating heterogeneity – total dairy.

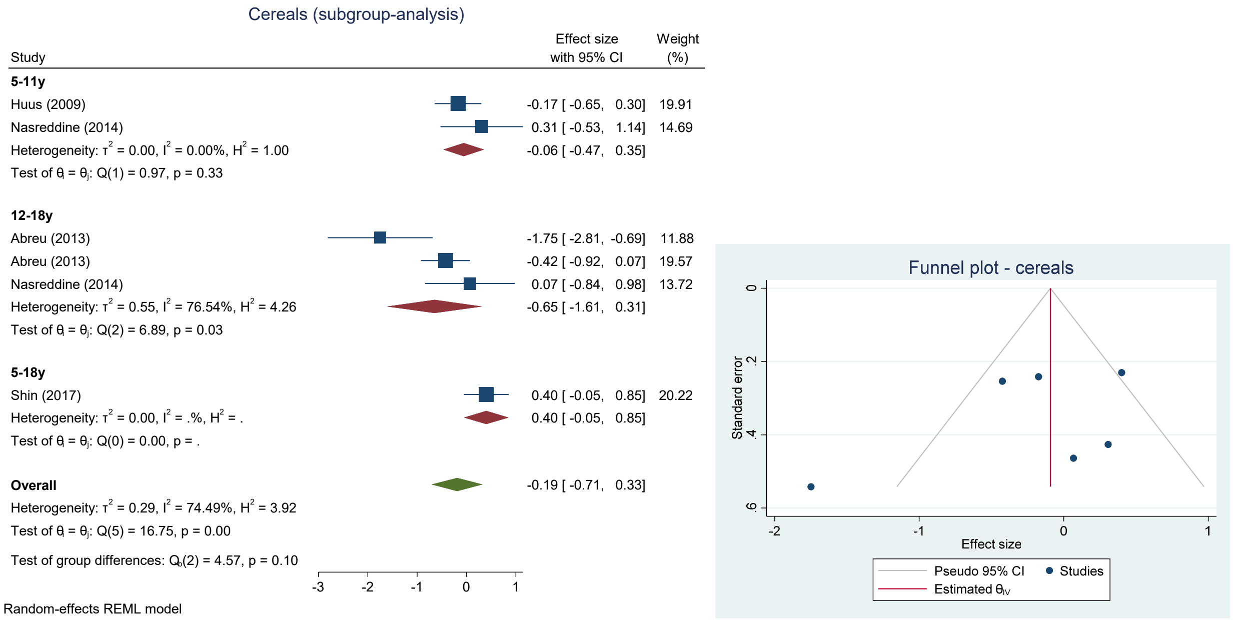


**Figure S33.** Subgroup analysis according to age and funnel plot investigating heterogeneity – cereals.

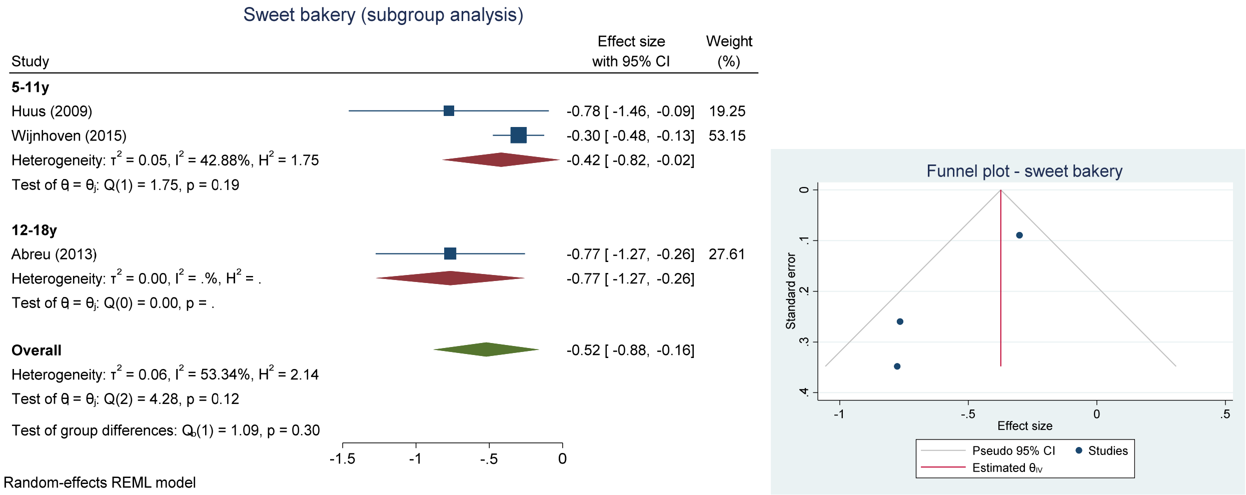


**Figure S34.** Subgroup analysis according to age and funnel plot investigating heterogeneity – sweet bakery.

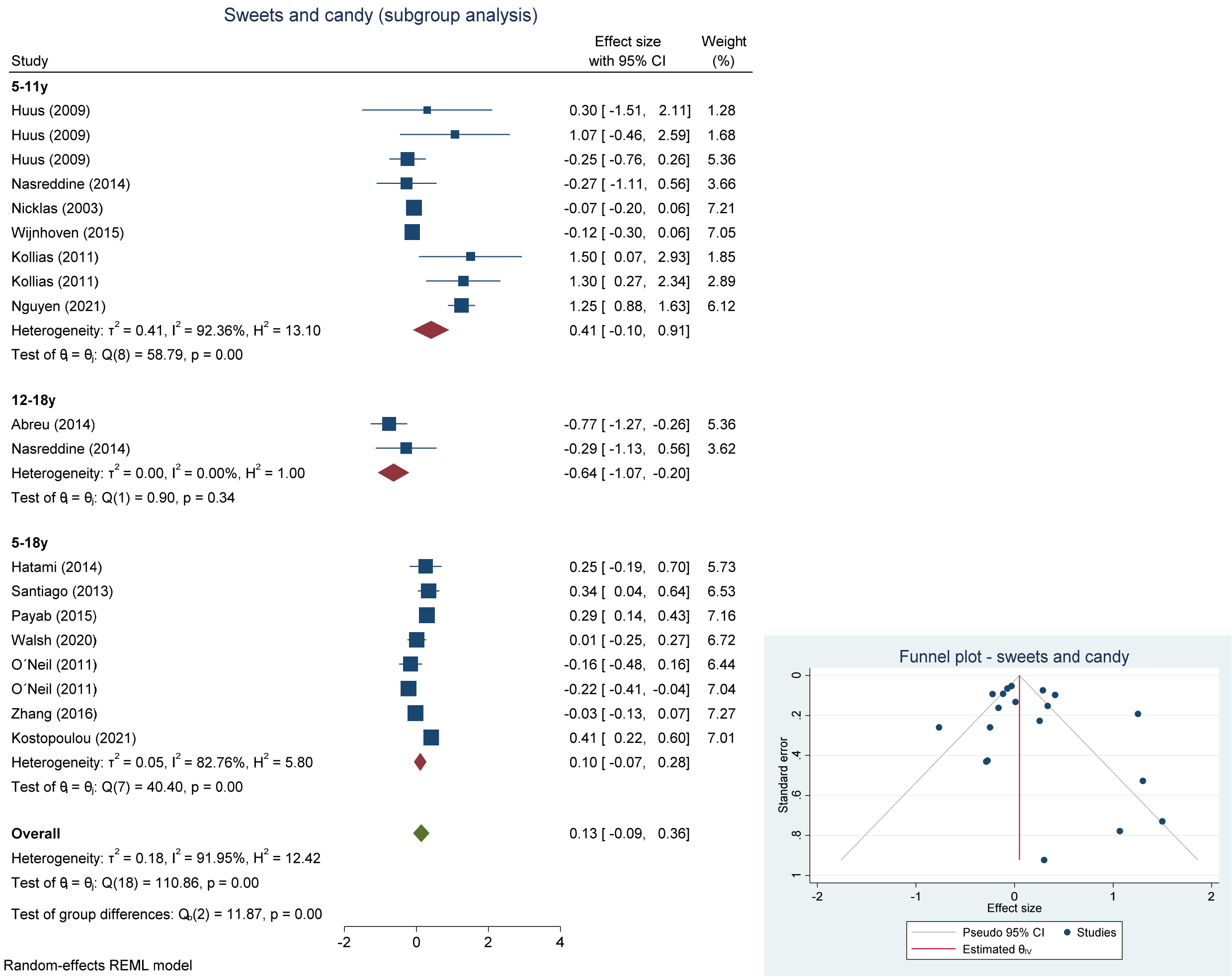


**Figure S35.** Subgroup analysis according to age and funnel plot investigating heterogeneity – sweets and candy.

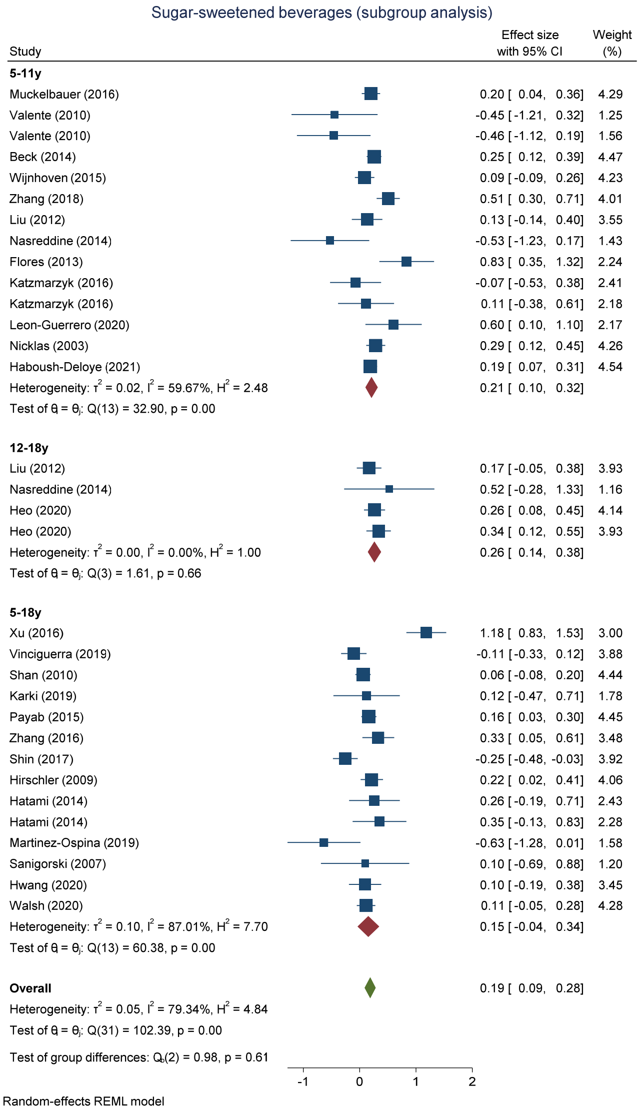


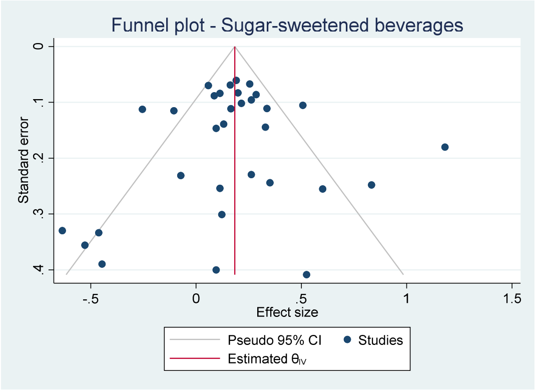


**Figure S36.** Subgroup analysis according to age and funnel plot investigating heterogeneity – sugar-sweetened beverages.

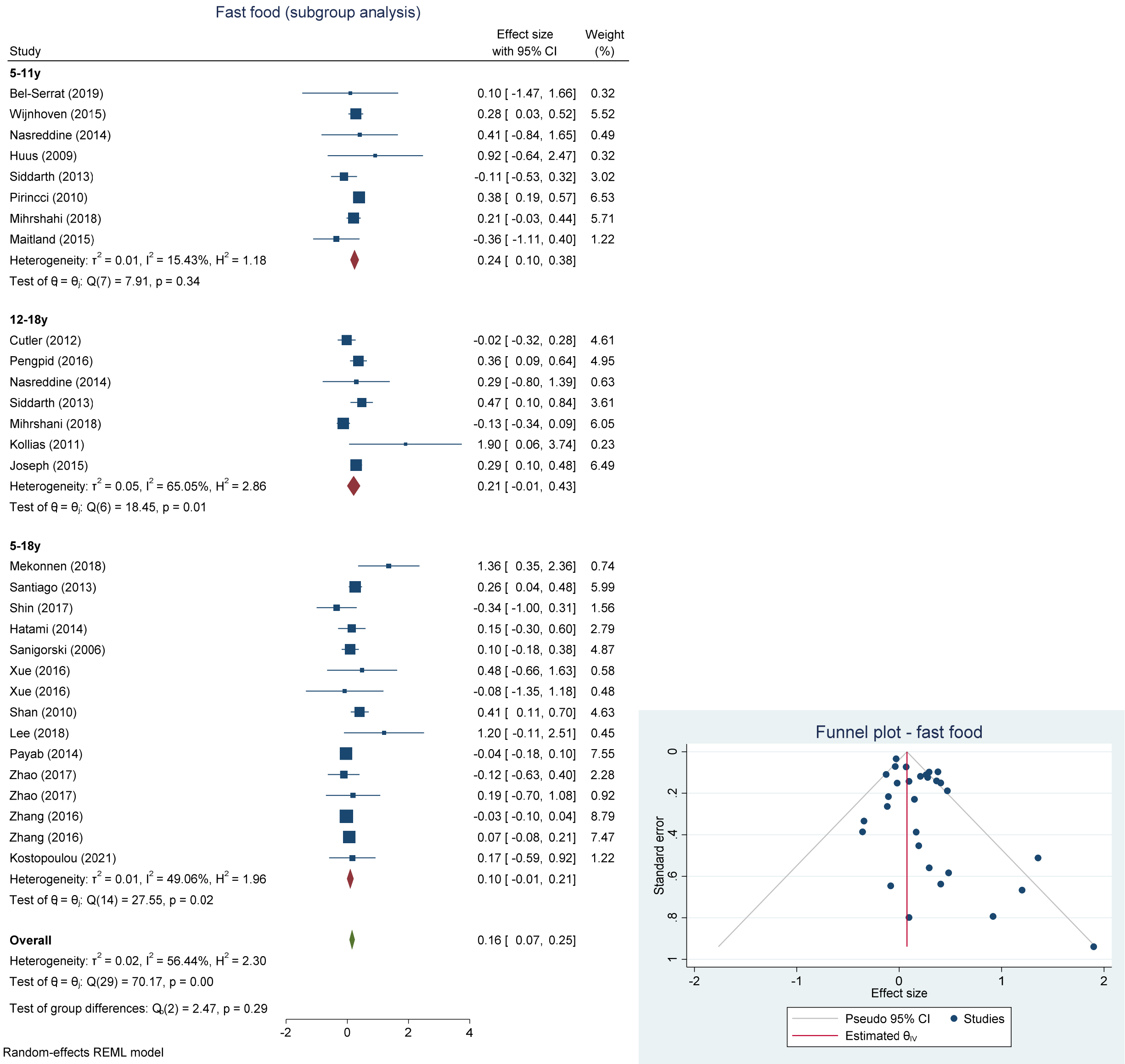


**Figure S37.** Subgroup analysis according to age and funnel plot investigating heterogeneity – fast food.

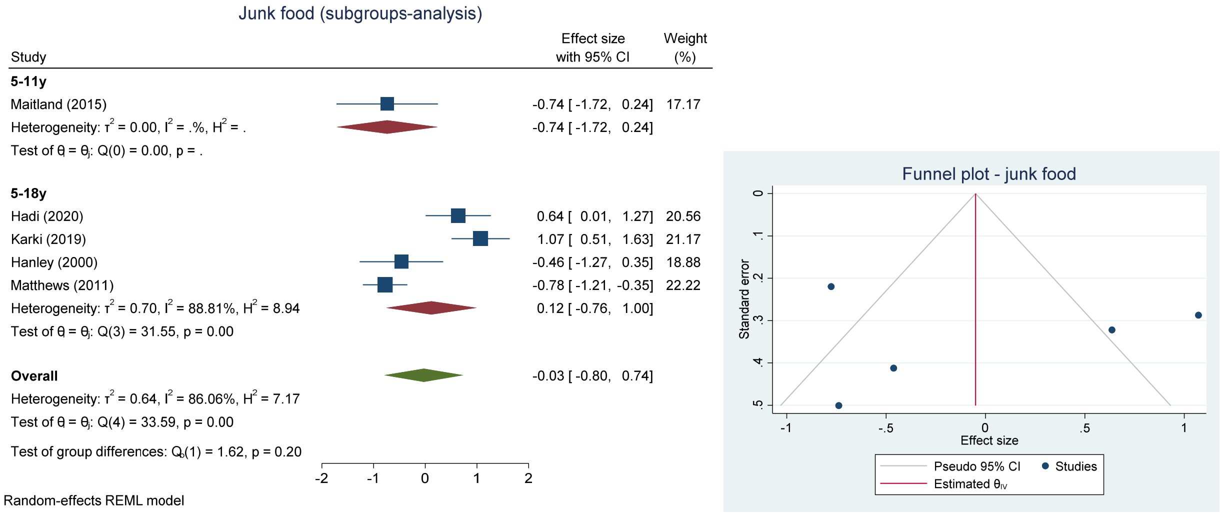


**Figure S38.** Subgroup analysis according to age and funnel plot investigating heterogeneity – junk food.

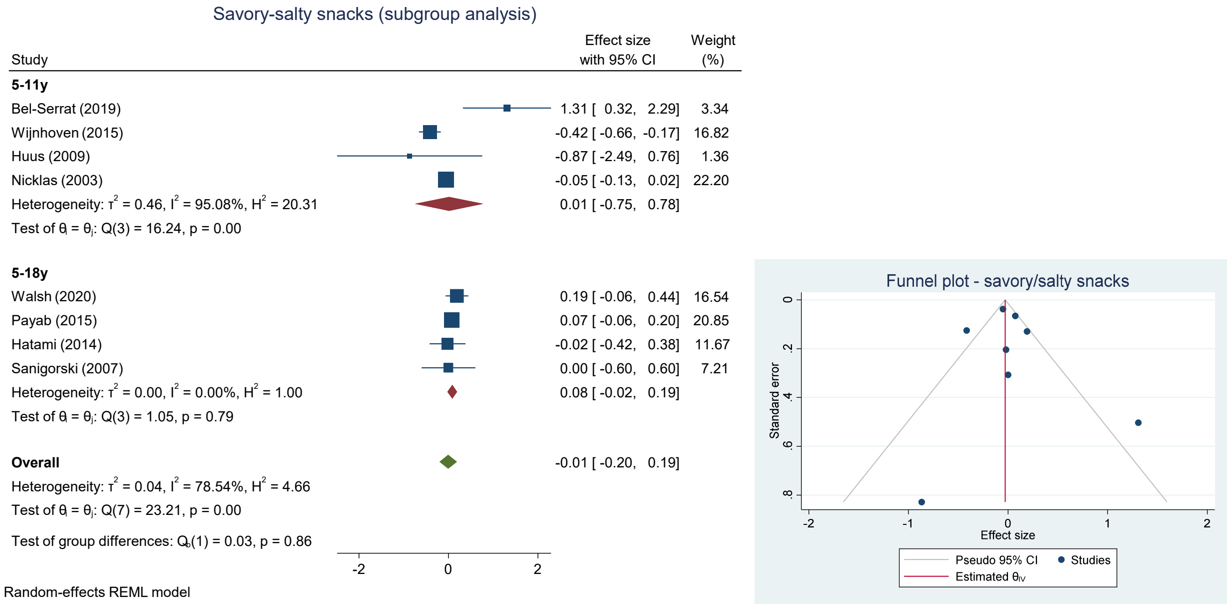


**Figure S39.** Subgroup analysis according to age and funnel plot investigating heterogeneity – savory-salty snacks.

## References


1. Abreu, S., et al., Food consumption, physical activity and socio-economic status related to BMI, waist circumference and waist-to-height ratio in adolescents. Public Health Nutr, 2014. **17**(8): p. 1834-49.
2. Ahmed, J., et al., Prevalence of and factors associated with obesity among Pakistani schoolchildren: a school-based, cross-sectional study. East Mediterr Health J, 2013. **19**(3): p. 242-7.
3. Beck, A.L., et al., Association of beverage consumption with obesity in Mexican American children. Public Health Nutr, 2014. **17**(2): p. 338-44.
4. Bel-Serrat, S., et al., Predictors of weight status in school-aged children: a prospective cohort study. Eur J Clin Nutr, 2019. **73**(9): p. 1299-1306.
5. Chen, J., et al., The relationship between socioeconomic status and childhood overweight/obesity is linked through paternal obesity and dietary intake: a cross-sectional study in Chongqing, China. Environ Health Prev Med, 2021. **26**(1): p. 56.
6. Choumenkovitch, S.F., et al., Whole grain consumption is inversely associated with BMI Z-score in rural school-aged children. Public Health Nutr, 2013. **16**(2): p. 212-8.
7. Colapinto, C.K., et al., Obesity, lifestyle and socio-economic determinants of vitamin D intake: a population-based study of Canadian children. Can J Public Health, 2014. **105**(6): p. e418-24.
8. Cutler, G.J., et al., Association between major patterns of dietary intake and weight status in adolescents. Br J Nutr, 2012. **108**(2): p. 349-56.
9. Denova-Gutiérrez, E., et al., Association between sweetened beverage consumption and body mass index, proportion of body fat and body fat distribution in Mexican adolescents. Ann Nutr Metab, 2008. **53**(3-4): p. 245-51.
10. Duan, R., et al., Prevalence and correlates of overweight and obesity among adolescents in northeastern China: a cross-sectional study. BMJ Open, 2020. **10**(7): p. e036820.
11. Flores, G. and H. Lin, *Factors predicting severe childhood obesity in kindergarteners.* Int J Obes (Lond), 2013. **37**(1): p. 31-9.
12. Gibson, S. and D. Neate, Sugar intake, soft drink consumption and body weight among British children: further analysis of National Diet and Nutrition Survey data with adjustment for under-reporting and physical activity. Int J Food Sci Nutr, 2007. **58**(6): p. 445-60.
13. Govindan, M., et al., Gender differences in physiologic markers and health behaviors associated with childhood obesity. Pediatrics, 2013. **132**(3): p. 468-74.
14. Haboush-Deloye, A., et al., Obesity in Early Childhood: Examining the Relationship among Demographic, Behavioral, Nutritional, and Socioeconomic Factors. Child Obes, 2021. **17**(5): p. 349-356.
15. Hadi, H., et al., Improved Understanding of Interactions between Risk Factors for Child Obesity May Lead to Better Designed Prevention Policies and Programs in Indonesia. Nutrients, 2020. **12**(1).
16. Hanley, A.J., et al., Overweight among children and adolescents in a Native Canadian community: prevalence and associated factors. Am J Clin Nutr, 2000. **71**(3): p. 693-700.
17. Hatami, M., et al., Dietary factors as the major determinants of overweight and obesity among Iranian adolescents. A cross-sectional study. Appetite, 2014. **82**: p. 194-201.
18. Heo, M. and J. Wylie-Rosett, Being obese versus trying to lose weight: Relationship with physical inactivity and soda drinking among high school students. J Sch Health, 2020. **90**(4): p. 301-305.
19. Hirschler, V., et al., Overweight and lifestyle behaviors of low socioeconomic elementary school children in Buenos Aires. BMC Pediatr, 2009. **9**: p. 17.

20. Huus, K., et al., Relationship of food frequencies as reported by parents to overweight and obesity at 5 years. Acta Paediatr, 2009. **98**(1): p. 139-43.
21. Hwang, S.B., et al., Trends in Beverage Consumption and Related Demographic Factors and Obesity among Korean Children and Adolescents. Nutrients, 2020. **12**(9).
22. Joseph, N., et al., Fast Food Consumption Pattern and Its Association with Overweight Among High School Boys in Mangalore City of Southern India. J Clin Diagn Res, 2015. **9**(5): p. Lc13-7.
23. Karki, A., A. Shrestha, and N. Subedi, Prevalence and associated factors of childhood overweight/obesity among primary school children in urban Nepal. BMC Public Health, 2019. **19**(1): p. 1055.
24. Katzmarzyk, P.T., et al., Relationship between Soft Drink Consumption and Obesity in 9-11 Years Old Children in a Multi-National Study. Nutrients, 2016. **8**(12).
25. Kollias, A., et al., Obesity and associated cardiovascular risk factors among schoolchildren in Greece: a cross-sectional study and review of the literature. J Pediatr Endocrinol Metab, 2011. **24**(11-12): p. 929-38.
26. Kostopoulou, E., et al., Association of lifestyle factors with a high prevalence of overweight and obesity in Greek children aged 10-16 years. Acta Paediatr, 2021. **110**(12): p. 3356-3364.
27. Lee, E.Y., et al., Study Time after School and Habitual Eating Are Associated with Risk for Obesity among Overweight Korean Children: A Prospective Study. Obes Facts, 2018. **11**(1): p. 46-55.
28. Leon Guerrero, R.T., et al., Prevalence and Predictors of Overweight and Obesity among Young Children in the Children's Healthy Living Study on Guam. Nutrients, 2020. **12**(9).
29. Liu, J.H., et al., Diet, physical activity, and sedentary behaviors as risk factors for childhood obesity: an urban and rural comparison. Child Obes, 2012. **8**(5): p. 440-8.
30. Maitland, T.E., S. Malcolm, and S. Handfield, Nutritional Knowledge and Practices, Lifestyle Characteristics and Anthropometric Status of Turks and Caicos Islands Elementary School Children. West Indian Med J, 2015. **64**(1): p. 29-36.
31. Marcos-Pasero, H., et al., Association of calcium and dairy product consumption with childhood obesity and the presence of a Brain Derived Neurotropic Factor-Antisense (BDNF-AS) polymorphism. Clin Nutr, 2019. **38**(6): p. 2616-2622.
32. Martinez-Ospina, A., et al., School Food Environment, Food Consumption, and Indicators of Adiposity Among Students 7-14 Years in Bogotá, Colombia. J Sch Health, 2019. **89**(3): p. 200-209.
33. Matthews, V.L., M. Wien, and J. Sabaté, The risk of child and adolescent overweight is related to types of food consumed. Nutr J, 2011. **10**: p. 71.
34. Mekonnen, T., A. Tariku, and S.M. Abebe, Overweight/obesity among school aged children in Bahir Dar City: cross sectional study. Ital J Pediatr, 2018. **44**(1): p. 17.
35. Mihrshahi, S., et al., Associations between childhood overweight, obesity, abdominal obesity and obesogenic behaviors and practices in Australian homes. BMC Public Health, 2017. **18**(1): p. 44.
36. Muckelbauer, R., et al., Changes in water and sugar-containing beverage consumption and body weight outcomes in children. Br J Nutr, 2016. **115**(11): p. 2057-66.
37. Nasreddine, L., et al., Dietary, lifestyle and socio-economic correlates of overweight, obesity and central adiposity in Lebanese children and adolescents. Nutrients, 2014. **6**(3): p. 1038-62.
38. Nguyen, T., et al., Early childhood factors associated with obesity at age 8 in Vietnamese children: The Young Lives Cohort Study. BMC Public Health, 2021. **21**(1): p. 301.
39. Nicklas, T.A., et al., Eating patterns and obesity in children. The Bogalusa Heart Study. Am J Prev Med, 2003. **25**(1): p. 9-16.
40. Notara, V., et al., Lack of association between dietary fibres intake and childhood obesity: an epidemiological study among preadolescents in Greece. Int J Food Sci Nutr, 2020. **71**(5): p. 635-643.
41. O'Neil, C.E., V.L. Fulgoni, 3rd, and T.A. Nicklas, Association of candy consumption with body weight measures, other health risk factors for cardiovascular disease, and diet quality in US children and adolescents: NHANES 1999-2004. Food Nutr Res, 2011. **55**.
42. Payab, M., et al., Association of junk food consumption with high blood pressure and obesity in Iranian children and adolescents: the CASPIAN-IV Study. J Pediatr (Rio J), 2015. **91**(2): p. 196-205.
43. Pengpid, S. and K. Peltzer, OVERWEIGHT, OBESITY AND ASSOCIATED FACTORS AMONG 13-15 YEARS OLD STUDENTS IN THE ASSOCIATION OF SOUTHEAST ASIAN NATIONS MEMBER COUNTRIES, 2007-2014. Southeast Asian J Trop Med Public Health, 2016. **47**(2): p. 250-62.
44. Pirinçci, E., et al., Prevalence and risk factors of overweight and obesity among urban school children in Elazig city, Eastern Turkey, 2007. Ann Hum Biol, 2010. **37**(1): p. 44-56.
45. Sakaki, J.R., et al., Associations between 100% Orange Juice Consumption and Dietary, Lifestyle and Anthropometric Characteristics in a Cross-Sectional Study of U.S. Children and Adolescents. Nutrients, 2019. **11**(11).
46. Sanigorski, A.M., A.C. Bell, and B.A. Swinburn, *Association of key foods and beverages with obesity in Australian schoolchildren.* Public Health Nutr, 2007. **10**(2): p. 152-7.
47. Santiago, S., et al., Gender differences in lifestyle determinants of overweight prevalence in a sample of Southern European children. Obes Res Clin Pract, 2013. **7**(5): p. e391-400.
48. Shan, X.Y., et al., Prevalence and behavioral risk factors of overweight and obesity among children aged 2-18 in Beijing, China. Int J Pediatr Obes, 2010. **5**(5): p. 383-9.
49. Shin, S.M., Association of Meat Intake with Overweight and Obesity among School-aged Children and Adolescents. J Obes Metab Syndr, 2017. **26**(3): p. 217-226.

50. Siddarth, D., *Risk factors for obesity in children and adults.* J Investig Med, 2013. **61**(6): p. 1039-42.
51. Valente, H., et al., Sugar-sweetened beverage intake and overweight in children from a Mediterranean country. Public Health Nutr, 2011. **14**(1): p. 127-32.
52. Vinciguerra, F., et al., Impact of unhealthy childhood and unfavorable parents' characteristics on adiposity in schoolchildren. Diabetes Metab Res Rev, 2019. **35**(8): p. e3199.
53. Walsh, C., et al., Snacking, sugar-sweetened beverage consumption and child obesity in low-income households. Nutrition & Food Science, 2020. 51(1): p. 151-163
54. White, M.J., et al., Associations between milk fat content and obesity, 1999 to 2016. Pediatr Obes, 2020. **15**(5): p. e12612.
55. Wijnhoven, T.M., et al., WHO European Childhood Obesity Surveillance Initiative: health-risk behaviours on nutrition and physical activity in 6-9-year-old schoolchildren. Public Health Nutr, 2015. **18**(17): p. 3108-24.
56. Xu, X., et al., Socioeconomic and lifestyle behavioral factors associated with overweight and obesity among rural to urban migrant children in central China. International Journal of Clinical and Experimental Medicine, 2016. **9**(11): p. 21635-21644.
57. Xue, H., et al., Time Trends in Fast Food Consumption and Its Association with Obesity among Children in China. PLoS One, 2016. **11**(3): p. e0151141.
58. Zhang, T., et al., The prevalence of obesity and influence of early life and behavioral factors on obesity in Chinese children in Guangzhou. BMC Public Health, 2016. **16**(1): p. 954.
59. Zhang, J., et al., Effect of Sleep Duration, Diet, and Physical Activity on Obesity and Overweight Elementary School Students in Shanghai. J Sch Health, 2018. **88**(2): p. 112-121.
60. Zhao, Y., et al., Fast food consumption and its associations with obesity and hypertension among children: results from the baseline data of the Childhood Obesity Study in China Mega-cities. BMC Public Health, 2017. **17**(1): p. 933.
